# Associations between various markers of intestinal barrier and immune function after a high‐intensity exercise challenge

**DOI:** 10.14814/phy2.16087

**Published:** 2024-05-23

**Authors:** Maria Fernanda Roca Rubio, Mattias Folkesson, Carolin Kremp, Niklas Evertsson, Dirk Repsilber, Ulrika Eriksson, John‐Peter Ganda Mall, Fawzi Kadi, Robert J. Brummer, Julia König

**Affiliations:** ^1^ Nutrition‐Gut‐Brain Interactions Research Centre, School of Medical Sciences, Faculty of Medicine and Health Örebro University Örebro Sweden; ^2^ Division of Sports Sciences, School of Health Sciences, Faculty of Medicine and Health Örebro University Örebro Sweden; ^3^ Man‐Technology‐Environment (MTM) Research Centre, School of Science and Technology Örebro University Örebro Sweden

**Keywords:** gastrointestinal symptoms, high‐intensity exercise, intestinal barrier function, intestinal permeability

## Abstract

Strenuous exercise can result in disruption of intestinal barrier function and occurrence of gastrointestinal symptoms. The aim of this exploratory study was to elucidate systemic effects of increased intestinal permeability after high‐intensity exercise. Forty‐one endurance‐trained subjects performed a 60‐min treadmill run at 80% VO_2_max. Small intestinal permeability was measured as urinary excretion ratio of lactulose/rhamnose (L/R). Blood, saliva and feces were analyzed for gut barrier and immune‐related biomarkers. The exercise challenge increased several markers of intestinal barrier disruption, immune function and oxidative stress. We found a negative correlation between L/R ratio and uric acid (*r* = −0.480), as well as a positive correlation between the L/R ratio and fecal chromogranin A in male participants (*r* = 0.555). No significant correlations were found between any of the markers and gastrointestinal symptoms, however, perceived exertion correlated with the combination of IL‐6, IL‐10 and salivary cortisol (*r* = 0.492). The lack of correlation between intestinal permeability and gastrointestinal symptoms could be due to minor symptoms experienced in lab settings compared to real‐life competitions. The correlation between L/R ratio and uric acid might imply a barrier‐protective effect of uric acid, and inflammatory processes due to strenuous exercise seem to play an important role regarding physical exhaustion.

## 1 INTRODUCTION

Physical activity has numerous beneficial effects on health. However, many athletes suffer from gastrointestinal problems due to strenuous physical exercise that negatively affect their performance and wellbeing (Peters et al., 1999; ter Steege et al., 2008). Emerging evidence has shown that strenuous physical exercise disrupts the intestinal barrier function and increases small intestinal permeability (Chantler et al., 2021; Costa et al., 2017; Engel et al., 2022; Keirns et al., 2020; March et al., 2017). It is suggested that the blood flow redistribution during strenuous exercise leads to intestinal hypoperfusion and hypoxia (splanchnic ischemia) which can cause intestinal damage (Qamar & Read, 1987; Schellekens et al., 2017; ter Steege et al., 2012; Van Wijck, Lenaerts, Van Bijnen, et al., 2012; van Wijck, Lenaerts, et al., 2011). In addition, the subsequent reperfusion after exercise can trigger the production of reactive oxygen species (ROS), resulting in oxidative stress further injuring the intestinal barrier, and potentially increasing intestinal permeability (Chantler et al., 2021; Costa et al., 2017; Lambert, 2004; Schellekens et al., 2017; Van Wijck, Lenaerts, Van Bijnen, et al., 2012; van Wijck, Lenaerts, et al., 2011). Increased permeability could then allow the translocation of microbial products and remnants into the portal blood stream, leading to a systemic proinflammatory immune response (Chantler et al., 2021; Costa et al., 2017; Lambert, 2004; Van Wijck, Lenaerts, Van Bijnen, et al., 2012; van Wijck, Lenaerts, et al., 2011). This cascade is hypothesized to be one of the contributing factors for the onset of gastrointestinal symptoms during exercise (Chantler et al., 2021; Costa et al., 2017), albeit studies investigating correlations between intestinal barrier function and symptoms have shown inconsistent results (Jeukendrup et al., 2000; Karhu et al., 2017; Pugh et al., 2017, 2019; van Nieuwenhoven et al., 2004; van Wijck, Lenaerts, et al., 2011). As strenuous exercise involves various other functional physiological processes, the link between these and markers of intestinal permeability requires further investigations.

In this exploratory study we assessed the effect of strenuous exercise on several markers of intestinal function and immune and inflammatory status, and evaluated the relationship among them using correlation analyses, with the aim to elucidate the systemic effects of an increased intestinal permeability after strenuous exercise. For this purpose, 41 endurance‐trained subjects with self‐reported gastrointestinal symptoms during exercise underwent an exercise challenge that consisted of 60 min treadmill running at the minimum speed required to achieve 80% maximal oxygen consumption (VO_2_ max). The control visit (no exercise) was performed 1 week before the exercise challenge, and on both visits, biomarker data were collected.

## 2 MATERIALS AND METHODS

### 2.1 Ethics statement

The study was conducted according to the principles of the Declaration of Helsinki and its revisions, and ethical approval was obtained from the Central Ethical Review Board of Uppsala, Sweden (registration number 2015/077, amendment 2022‐02024‐02). The study was part of a larger unpublished intervention study performed at Örebro University in Örebro, Sweden from September 2015 until January 2016. All participants were recruited from the greater area of Örebro and gave their written informed consent before participation. The trial was registered at www.isrctn.com (ISRCTN16686476) on November 6, 2015.

### 2.2 Sample size calculations

Sample size calculations were performed as part of a larger intervention study in which the primary endpoint was to detect differences in small intestinal permeability measured as urinary lactulose/rhamnose excretion (L/R ratio) between one of two probiotic study arms and the placebo arm. Based on previous data regarding the effect of strenuous exercise on intestinal permeability by Zuhl et al. 2016 (relevant change of 20% in L/R ratio, amounting to 0.012 points in ratio difference, SD of 0.01), it was estimated that with a power of 80% and a 95% confidence interval, and an expected drop‐out rate of 10%, when using a two‐sided Mann–Whitney *U*‐test for independent samples, *n* = 16 subjects needed to be included in each arm, that is, *n* = 48 subjects in total. In this study, the available number of samples (*n* = 41) allowed for a Cohen's d (i.e., ratio of detectable difference and SD) of 0.6, which can be considered a moderate effect size for all the investigated markers.

### 2.3 Participants

Female and male endurance‐trained subjects self‐reporting gastrointestinal complaints when exercising, but otherwise healthy, were recruited by advertisements in the greater area of Örebro. After having given their written informed consent, subjects completed screening procedures to evaluate their eligibility for the study. Inclusion criteria were to be healthy apart from gastrointestinal symptoms, the presence of upper or lower gastrointestinal symptoms that interfere with training and during competition and might also be present at rest, age between 18 and 40 years, a weekly training load of 4 or more hours within endurance sports (minimum 50% of the training should be running activity), ability to complete a 10‐km run within 60 min, and willingness to abstain from any probiotic products or medication known to alter gastrointestinal function throughout the study. Exclusion criteria were abdominal surgery which might affect gastrointestinal function (except for appendectomy and cholecystectomy), high blood pressure (≥90 mmHg/140 mmHg), current diagnosis of psychiatric disease, systemic use of antibiotics or steroids or antimicrobial medication during the 4 months prior to screening, daily usage of nonsteroidal anti‐inflammatory drugs (NSAIDs) 2 months or incidental use 2 weeks prior to screening, usage of medications (except oral contraceptives) 2 weeks prior to screening, diagnosed inflammatory gastrointestinal disease, lactose intolerance, any disease which could interfere with the intestinal barrier function, participation in other clinical trials 3 months prior to screening, regular use of probiotics 2 months prior to screening, smoking and/or use of chewable tobacco, planned change to current diet or exercise regime, use of laxatives, anti‐diarrhoeal and anti‐cholinergic medications 2 months prior to screening, use of immunosuppressant drugs 4 weeks prior to screening, and pregnancy or breastfeeding.

### 2.4 Study design

In this study, a total of 46 endurance‐trained subjects were recruited of which 5 dropped out due to personal reasons or illness. Participants were asked to attend three visits: (1) VO_2_ max test on a treadmill, (2) control visit, and (3) exercise challenge. Visit 1 and visit 2 were separated by a period of 2 weeks, while visit 2 and visit 3 were separated by 1 week. Visit two and three were performed at similar hours of the day to avoid diurnal hormonal changes. During the entire study period the subjects were asked not to consume any probiotic products as well as to maintain their habitual diet and lifestyle such as physical activity level and sleep habits. Subjects were asked to record their food and fluid intake 2 days before visit two, and then replicate this diet 2 days before visit 3. In addition, participants were asked to refrain from consuming alcohol, caffeine, spicy foods, and artificial sugars as well as to avoid strenuous exercise 2 days prior to each visit. The evening before each visit, the subjects consumed a standardized energy adjusted dinner and an evening snack provided by the study team and fasted overnight except for a standardized energy adjusted breakfast prior to all biological samplings. Water intake (ad libitum) was allowed.

### 2.5 Maximal oxygen consumption (VO_2_ max) test

During visit 1, an incremental test was performed to measure VO_2_ max and subsequently establish the minimum running velocity required to achieve a VO_2_ max of 80%. The test was performed on a motorized treadmill (Rodby RL 2500, Rodby Innovation AB, Sweden) with an initial speed of 10 km/h. Treadmill speed was then increased with 1.0 km/h every 2 min until subjects achieved volitional exhaustion (Billat et al., 1996). During the entire test treadmill inclination was set at 1% (Jones & Doust, 1996). In addition, heart rate and respiratory gases were measured during the test (Jaeger Oxycon Pro, Carefusion, Germany).

### 2.6 Control visit

At the control visit (visit 2), the sugar solution was administered at the estimated time of its administration at visit 3, followed by total urinary collection for 5 h. Also, saliva and blood samples were collected at an estimated similar time point as during visit 3. Fecal samples from the first bowel movement after intake of the sugar solution were collected by each subject using the EasySampler device (GP Medical Devices ApS, Denmark). Samples were either delivered directly to the study staff or stored in a home freezer (−18°C or below) until transport in special cool transport containers (Sarstedt, Germany) to the study centre.

### 2.7 Exercise challenge to induce increased intestinal permeability

During visit 3, participants completed an exercise test to induce increased intestinal permeability. The exercise test was performed as a 60‐min treadmill run at a velocity corresponding to 80% of VO_2_ max. If a participant was not able to run the complete 60 min at the speed corresponding to 80% of VO_2_ max, the speed was reduced to 70%, and, if necessary, to 60% of VO_2_ max. In case of reduced speed, the running time was extended so that the participants covered the same distance as if the run had been performed at the velocity corresponding to 80% of VO_2_ max. Every 5 min during the exercise test, subjects were asked for perceived local and central exertion according to Borg's rating of perceived exertion scale (scale from to 20) (Borg, 1998). The average temperature of the test room was 21.9 ± 0.3°C with a relative humidity of 46.1 ± 3.1%. After completion of 30 min of the 60‐min exercise test, participants drank the multi‐sugar solution, followed by total urinary collection for 5 h. After the exercise challenge, participants were asked about gastrointestinal symptoms using a Likert‐scale. Within 10 min after the exercise test, venous blood samples were collected. Saliva samples were collected 25 min after the exercise test. Fecal samples from the first bowel movement after intake of the sugar solution were collected following the same protocol as at visit 2.

### 2.8 Markers of gut barrier function

#### 2.8.1 L/R urinary recovery test

This small intestinal permeability test was performed according to van Wijck et al (van Wijck, van Eijk, et al., 2011). 150 mL of a tap water solution containing 1.0 g of lactulose (Solactis, France) and 0.5 g of L‐rhamnose (product number 117813‐518, Biogaia, Sweden) were orally administered, followed by 5 h of urine collection. During these 5 h, subjects refrained from food intake and were asked to drink at least 1.5 L of water. Urine was collected by the subjects in provided collection jars (Sarstedt, Sweden) and stored in cooling bags with cooling elements. After finalizing the urine collection, subjects delivered it to the university staff. Upon delivery, 1 mL of urine was centrifuged at 21,382 × *g* for 25 min at a temperature of 4°C. The supernatant was collected and stored at −80°C until further analyses by ultra‐performance liquid chromatography–tandem mass spectrometry (UPLC‐MS/MS) as previously described (Ganda Mall et al., 2020).

#### 2.8.2 Intestinal fatty acid‐binding protein (I‐FABP)

Heparin plasma samples were analyzed for plasma levels of I‐FABP using an enzyme‐linked immunosorbent assay (ELISA) kit (HK406, Hycult Biotech, the Netherlands) according to the manufacturer's instructions.

#### 2.8.3 16S ribosomal ribonucleic acid (16S rRNA)

Quantification and sequencing of bacterial 16S rRNA gene fragments in whole blood was performed by Vaiomer (France) according to Paisse et al. (2016).

#### 2.8.4 Lipopolysaccharides (LPS)

LPS endotoxin concentrations were assessed in serum samples by Vaiomer, France, using the Limulus Amebocyte Lysate (LAL) kinetic chromogenic methodology according to the manufacturer's instructions.

### 2.9 Assessment of immune function

#### 2.9.1 Fecal calprotectin

Fecal calprotectin was measured using an ELISA kit (K6927, Immundiagnostik AG, Germany) according to the manufacturer's instructions.

#### 2.9.2 High sensitivity C‐reactive protein (hs‐CRP)

Hs‐CRP analysis was performed in plasma samples according to clinical routine by the Department of Laboratory Medicine at Örebro University Hospital. Hs‐CRP concentrations were measured by a latex‐enhanced immunoturbidimetric assay (ADVIA® Chemistry CardioPhase™ High Sensitivity C‐Reactive Protein, Siemens Healthcare Diagnostics Inc, Germany). The detection range was specified as 0.16 to 10 mg/L, the intra‐assay variation as 0.8%–5.3% and the inter‐assay variation as 0.9%–6.8%.

#### 2.9.3 Cytokines (IL‐6, IL‐8, IL‐10)

Cytokine analysis was performed by flow cytometric analysis of serum samples using the Human Inflammatory cytokine Cytometric Bead Array (551811 BD™, BD Biosciences, U.S.A.) according to the manufacturer's instructions.

### 2.10 Markers of oxidative stress

#### 2.10.1 Protein carbonyls

Protein‐bound carbonyls in serum were measured using an ELISA kit (K7870, Immundiagnostik AG, Germany) according to the manufacturer's instructions.

#### 2.10.2 Uric acid

After collection, blood samples were transferred to microcentrifuge tubes and centrifuged at 15,000 RFC for 1 min at 4°C. After centrifugation, 400 mL of plasma were immediately mixed with 400 mL of MPA solution (10% meta‐phosphoric acid w/v containing 2 mM Na2EDTA). After vortexing, the samples were centrifuged again (15,000 RFC, 1 min at 4°C) and the supernatant was stored at −80°C until further analysis. Uric acid analysis was performed by high‐performance liquid chromatography (HPLC) at the University of Copenhagen, as previously described by Lykkesfeldt (2000).

### 2.11 Other markers

#### 2.11.1 Creatine kinase

Creatine kinase in plasma was assessed as a marker of muscle injury according to clinical routine by the Department of Laboratory Medicine at Örebro University Hospital.

#### 2.11.2 Plasma myeloperoxidase

Heparin plasma samples were analyzed for plasma levels of myeloperoxidase as a marker of neutrophil degranulation using an ELISA kit (HK324, Hycult Biotech) according to the manufacturer's instructions.

#### 2.11.3 Fecal human beta‐defensin 2

The antimicrobial peptide human beta‐defensin 2 (HBD2) was measured in stool samples using an ELISA kit (K6500, Immundiagnostik AG, Germany) according to the manufacturer's instructions.

#### 2.11.4 Fecal chromogranin A

Fecal chromogranin A was assessed as a potential gut‐brain marker using a commercial radioimmunoassay (RB 321, Euro Diagnostica AB, Malmö, Sweden) according to the manufacturer's instructions.

#### 2.11.5 Salivary secretory IgA and cortisol

Fifteen minutes after the end of the exercise challenge, the subjects were asked to stop drinking for 10 min to avoid dilution of saliva samples. Twenty‐five minutes after the end of the exercise challenge, subjects were asked to swallow to empty their mouth followed by an unstimulated whole saliva sampling by expectoration into 4 mL sampling vials. After collection, samples were stored at −80°C until further analyses. For the analyses, samples were thawed and then centrifuged at 3000 RCF for 15 min. Salivary soluble IgA (sIgA) was measured using an ELISA kit (1‐1602, Salimetrics, U.S.A.) according to the manufacturer's instructions. Salivary cortisol was measured using an ELISA kit (1‐3102, Salimetrics) according to the manufacturer's instructions.

### 2.12 Gastrointestinal symptoms rating scale (GSRS)

At baseline participants were asked to complete the GSRS to assess their gastrointestinal symptoms independent of exercise. The GSRS includes 15 symptoms and uses a 7‐point Likert scale in which 1 represents no discomfort at all and 7 very severe discomfort (Kulich et al., 2008).

### 2.13 Gastrointestinal symptoms rating after exercise

After the exercise challenge, participants were asked to rate gastrointestinal symptoms commonly reported by runners on a Likert scale ranging from 0 to 7 (0—no symptoms; 1—very mild; 2–4—mild; 4–6—moderate; 7—severe).

### 2.14 Data analysis

Normality of the data sets was tested with Shapiro–Wilk test. As most of the data was not normally distributed, differences among biomarker data between the exercise challenge and the control visit was tested by paired Wilcoxon signed‐rank tests, and sex differences were assessed by the Mann–Whitney *U*‐test. For the correlation analysis, biomarker data were analyzed using two‐tailed Spearman's rank‐order correlation. *p* < 0.05 were corrected for multiple comparisons by Benjamin‐Hochberg procedure with a false discovery rate (FDR) cut‐off at <5% (*q* < 0.05). Statistical calculations were performed with GraphPad Prism 9.3.1 (GraphPad Software Incorporated, La Jolla, CA, USA).

Due to the high number of variables in comparison to sample size, sparse canonical correlation analysis (sCCA) was used to correlate biomarkers with symptoms as a multi‐variable analysis. sCCA is a method that estimates the correlation between two sets of variables combined in a way that maximizes this correlation (Rousu et al., 2013). For this analysis, one data set included all the biomarker variables, while the second data set included gastrointestinal symptoms and perceived exertion after the exercise challenge. Exertion data used for the sCCA analysis were the Borg RPE scale values reported at 60 min of the exercise challenge, as well as the mean of all the values reported every 5 min. All data were log2‐transformed and standardized prior to sCCA analysis. sCCA was performed with R studio version 4.1.0 and R‐package “PMA”, therein function “CCA” (Witten et al., 2009). FDR estimates were calculated according to Storey (2002).

## 3 RESULTS

Endurance‐trained subjects with reported gastrointestinal complaints while exercising, but otherwise healthy, were included in this study (11 female and 30 males, mean age 29.1 ± 6.2 years). Characteristics of all participants are shown in Table 1. The average perceived central and local exertion during the exercise test were 14.8 for both, indicating a strenuous exercise challenge (Table 1).

**TABLE 1:** Characteristics of study participants.

	All (41)	Females (11)	Males (30)
Age (years)	29.1 ± 6.2	25.5 ± 5.4	30.4 ± 6.1
Body mass index (kg/m^2^)	23.1 ± 2.4	20.5 ± 1.5	24.0 ± 1.9
GSRS total scores at baseline	2.2 ± 0.5	2.3 ± 0.5	2.2 ± 0.5
Total training load (h/week)	6.6 ± 2.5	6.3 ± 2.6	6.8 ± 2.5
VO_2_ max (mL/kg/min)	53.1 ± 5.2	49.5 ± 4.6	54.4 ± 4.9
Completed challenge at 80%/70%/60% VO_2_ max (n)	13/22/6	2/8/1	11/14/5
Average time to complete challenge (min)	65.5 ± 4.7	64.5 ± 3.3	65.8 ± 5.1
Average speed during challenge (km/h)	11.1 ± 1.3	10.3 ± 1.1	11.4 ± 1.2
Average central exertion (RPE)	14.8 (14.0–15.6)	14.6 (13.6–15.4)	15.1 (14.0–15.6)
Central exertion at 60 min (RPE)	16.0 (15.0–17.8)	16.0 (15.0–17.0)	16.0 (15.0–18.0)
Average local exertion (RPE)	14.8 (13.5–15.8)	14.8 (12.2–15.8)	14.9 (14.0–15.8)
Local exertion at 60 min (RPE)	17.0 (15.5–18.0)	16.0 (15.0–17.0)	16.0 (15.0–18.0)

*Note*: Data are expressed as mean ± standard deviation or median (interquartile range) for parametric and non‐parametric data, respectively.

Abbreviations: GSRS, Gastrointestinal symptoms rating score; RPE, Borg's rating of perceived exertion; VO_2_ max, maximal oxygen consumption.

### 3.1 Gastrointestinal symptoms after the exercise challenge

All participants reported gastrointestinal complaints after the exercise challenge, mostly of very mild or mild severity. The most common symptoms were stitches (reported by 54%), belching (reported by 51%), abdominal pain (reported by 39%) and nausea (reported by 36%) (Table S1).

### 3.2 Effect of exercise on markers of intestinal barrier function, immune function, oxidative stress, and exploratory markers

After the exercise challenge, we found a significant increase in urinary L/R ratio, I‐FABP, 16S rRNA, myeloperoxidase, IL‐6, IL‐8, IL‐10, uric acid, and salivary cortisol. Fecal chromogranin A, LPS, fecal calprotectin, hs‐CRP, protein carbonyls, fecal HBD2, creatine kinase and salivary sIgA were not significantly affected (Table 2). If analyzed separately for female and male participants, only concentrations of uric acid differed significantly between them. They were significantly higher in males than in females both at the control visit (females: 146.2 (138.5–200.7), males: 260.2 (218.5–282.7), *p* < 0.0001; *q* = 0.002) as well as after the exercise challenge (females: 213.7 (200.1–223.0), males: 293.4 (255.2–313.6), *p* < 0.0001; *q* = 0.002) (Table S2).

**TABLE 2:** Levels of markers related to intestinal barrier function, immune response, oxidative stress, and exploratory markers at the control visit and after exercise.

Marker	Control	Post‐exercise	*p*‐value	*q*‐value
Urinary L/R ratio	0.031 (0.023–0.045)	0.046 (0.035–0.067)	<0.0001	**0.0054**
I‐FABP (pg/ml)	385 (240–531)	1784 (1057‐2765)	<0.0001	**0.0027**
16S rRNA (copies 16S/ng DNA)	50.8 (44.9–57.1)	55.5 (49.9–64.7)	<0.0001	**0.0018**
*Bacteroidota*/total 16S rRNA	0.048 (0.001–0.101)	0.002 (0.000–0.072)	0.051	
LPS (EU/mL)	0.084 (0.056–0.109)	0.083 (0.055–0.140)	>0.9999	
Fecal calprotectin (μg/mL)	27 (21–48)	28 (20–54)	0.307	
IL‐6 (pg/mL)	0.82 (0.82–0.822)	2.15 (0.82–4.59)	<0.0001	**0.0014**
IL‐8 (pg/mL)	4.82 (3.26–5.56)	6.94 (5.43–10.96)	<0.0001	**0.0011**
IL‐10 (pg/mL)	0.82 (0.82–0.82)	4.76 (2.91–10.67)	<0.0001	**0.0009**
hs‐CRP (mg/L)	0.52 (0.29–1.03)	0.55 (0.25–1.19)	0.850	
Myeloperoxidase (ng/mL)	26.4 (22.3–31.5)	37.1 (31.7–48.0)	<0.0001	**0.0008**
Protein carbonyls (U/mL)	161.1 (136.8–201.3)	179.7 (144.3–208.0)	0.075	
Uric acid (μmol/L)	227.8 (186.0–269.5)	285.3 (226.3–309.4)	<0.0001	**0.0007**
Fecal HBD2 (ng/mL)	54.3 (33.5–102.5)	47.7 (29.4–73.1)	0.952	
Fecal chromogranin A (nmol/g)	0.200 (0.145–0.525)	0.260 (0.160–0.740)	0.043	0.232
Creatine kinase (μkat/L)	2.0 (1.3–3.6)	2.6 (1.7–4.15)	0.136	
Salivary sIgA (μg/mL)	106.6 (83.7–168.4)	124.1 (90.1–179.1)	0.141	
Salivary cortisol (μg/dL)	0.258 (0.170–0.371)	1.102 (0.788–1.297)	<0.0001	**0.0006**

*Note*: Median and interquartile ranges (IQR) are shown. Differences between the two conditions were assessed by paired Wilcoxon signed‐rank test. The Benjamin‐Hochberg procedure with a false discovery rate FDR of <5% was used to corrected for multiple comparisons. Q‐values smaller than 0.05 indicate significant differences and are marked in bold.

Abbreviations: 16S rRNA, 16S ribosomal RNA gene copy numbers; HBD2, human ß‐defensin 2; hs‐CRP, high sensitivity C‐reactive protein; IL, interleukin; I‐FABP, intestinal fatty acid‐binding protein; LPS, lipopolysaccharides; sIgA, soluble immunoglobulin A; Urinary L/R, lactulose/rhamnose excretion ratio.

### 3.3 Correlations between markers of intestinal barrier function

**FIGURE 1:**
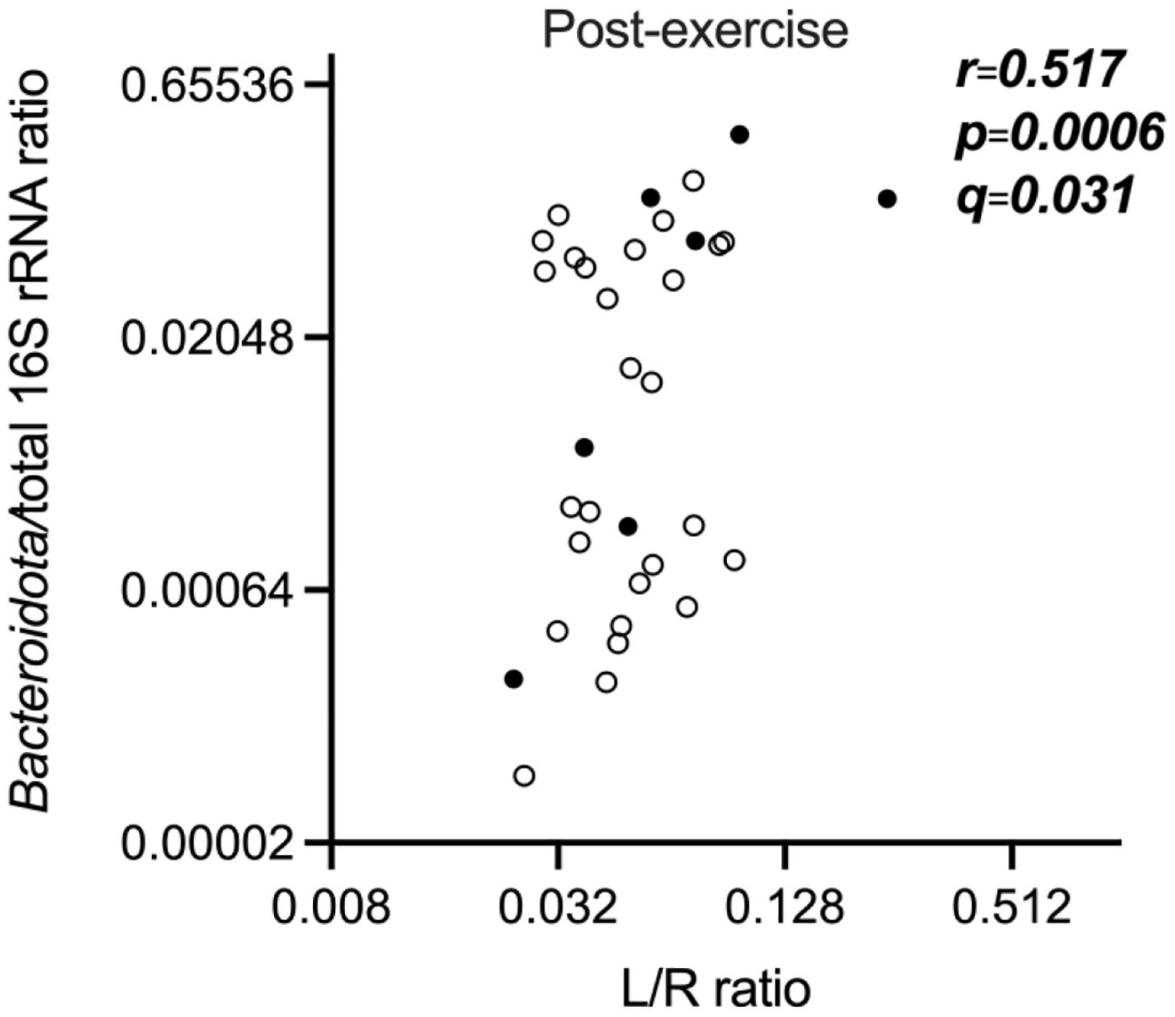
Scatter plot of the correlation between *Bacteroidota*/total 16S rRNA ratio and L/R ratio after the exercise challenge. Black dots represent female participants and open dots represent male participants. The analysis was performed by a two‐tailed Spearman's correlation analysis and corrected for multiplicity by the Benjamin‐Hochberg procedure if *p* < 0.05. *q*‐values smaller than 0.05 were considered statistically significant. The graph is shown in log‐scale to improve visualization. Three female and three male participants had too low values of *Bacteroidota*/total 16S rRNA ratio and are thus not shown in the graph. For one female participant, not enough blood samples were available for 16S rRNA analysis. L/R ratio – urinary lactulose/rhamnose excretion ratio.

No significant correlations were found among any of the markers associated with intestinal barrier function (L/R, I‐FABP, 16S rRNA, *Bacteroidota*/total 16S rRNA, LPS) at the control visit (Table S3). However, after the exercise challenge we observed a significant correlation between the *Bacteroidota*/total 16S rRNA ratio and L/R ratio in all participants (*r* = 0.517, *p* = 0.0006, *q* = 0.03; Figure 1, Table S4).

### 3.4 Correlations between markers of intestinal barrier function and markers of inflammatory immune response and oxidative stress

**FIGURE 2:**
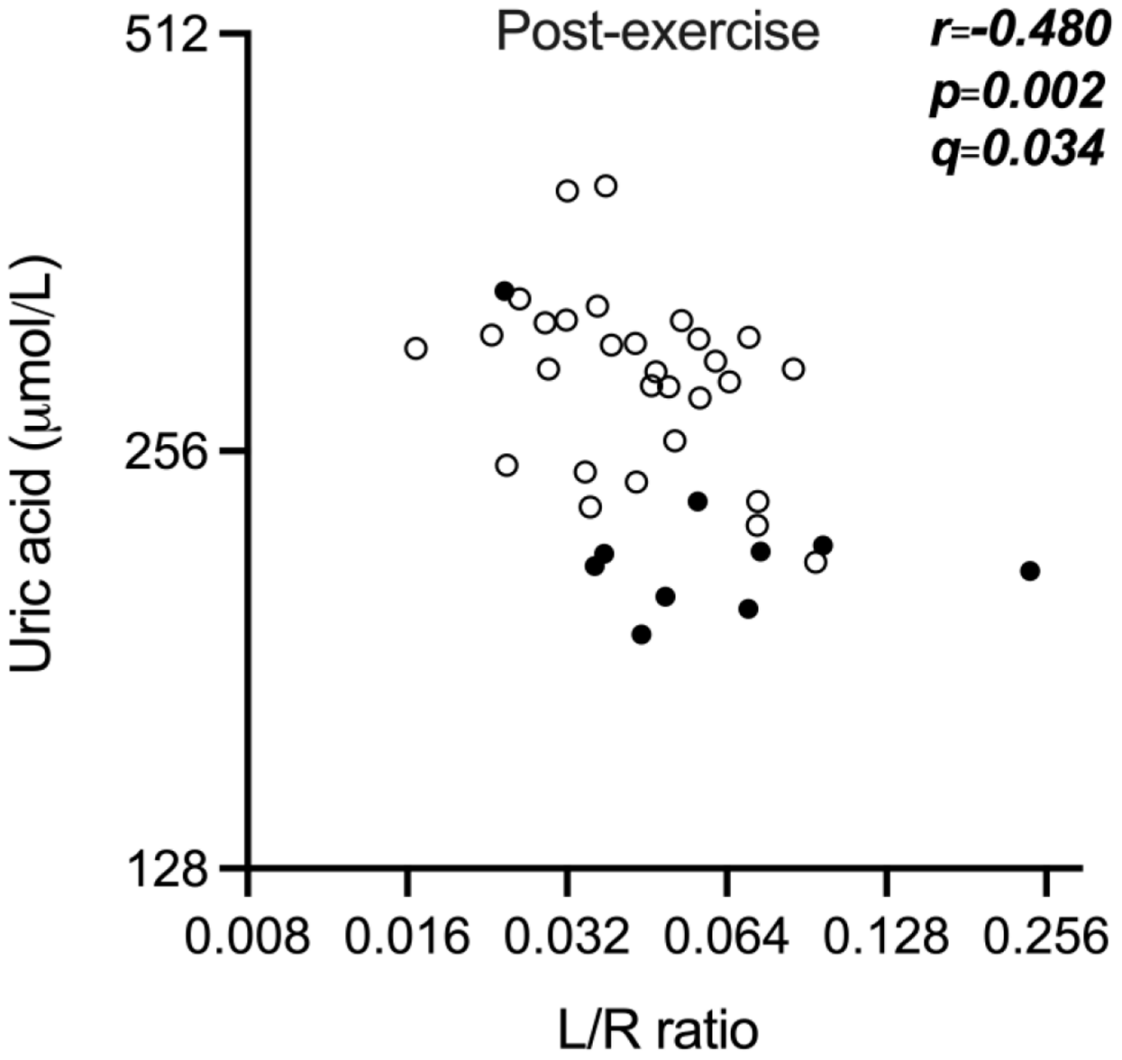
Scatter plot of the negative correlation between uric acid and L/R ratio after the exercise challenge. Black dots represent female participants and open dots represent male participants. The analysis was performed by a two‐tailed Spearman's correlation analysis and corrected for multiplicity by the Benjamin‐Hochberg procedure if *p* < 0.05. *q*‐values smaller than 0.05 were considered statistically significant. The graph is shown in log scale to improve visualization. One female and one male participant had missing values. L/R ratio—urinary lactulose/rhamnose excretion ratio.

We did not find any significant correlations at the control visit (Table S5). After the exercise challenge we found a significant negative correlation between the L/R ratio and uric acid in all participants (*r* = −0.480, *p* = 0.002, *q* = 0.048; Figure 2, Table S6).

### 3.5 Correlations between markers of intestinal barrier function and exploratory markers

**FIGURE 3:**
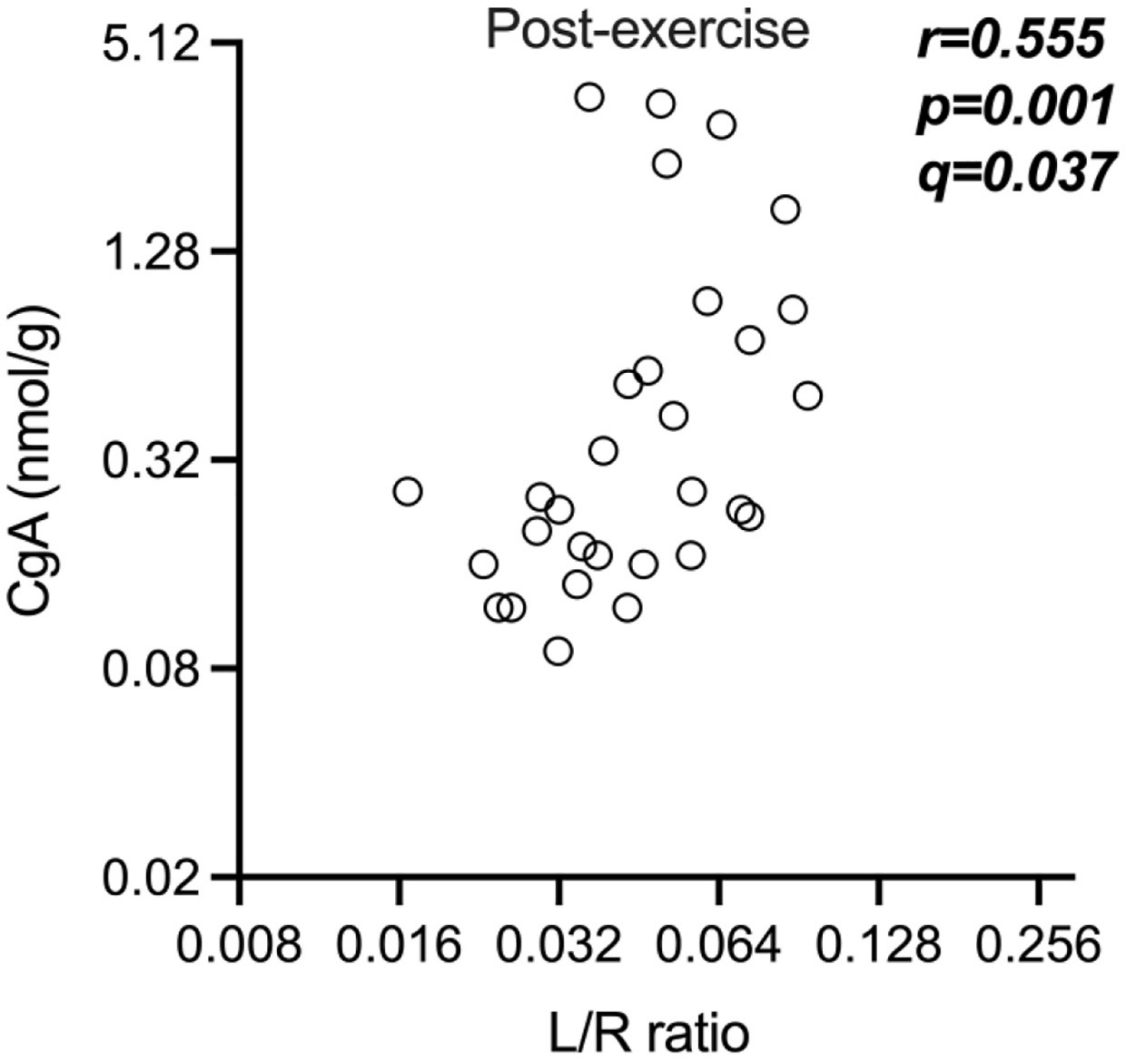
Scatter plot of the correlation observed in males between fecal chromogranin A (CgA) and L/R ratio after the exercise challenge. The analysis was performed by a two‐tailed Spearman's correlation analysis and corrected for multiplicity by the Benjamin‐Hochberg procedure if *p* < 0.05. *q*‐values smaller than 0.05 were considered statistically significant. The graph is shown in log scale to improve visualization. L/R ratio – urinary lactulose/rhamnose excretion ratio.

No correlations were found between the markers associated with intestinal barrier function and the exploratory markers at the control condition (Table S7). However, after the exercise challenge, we observed a significant correlation in male participants between fecal chromogranin A and the L/R ratio (*r* = 0.555, *p* = 0.001, *q* = 0.037; Figure 3, Table S8).

### 3.6 Correlations between biomarker data and reported gastrointestinal symptoms and perceived exertion after the exercise challenge

**FIGURE 4:**
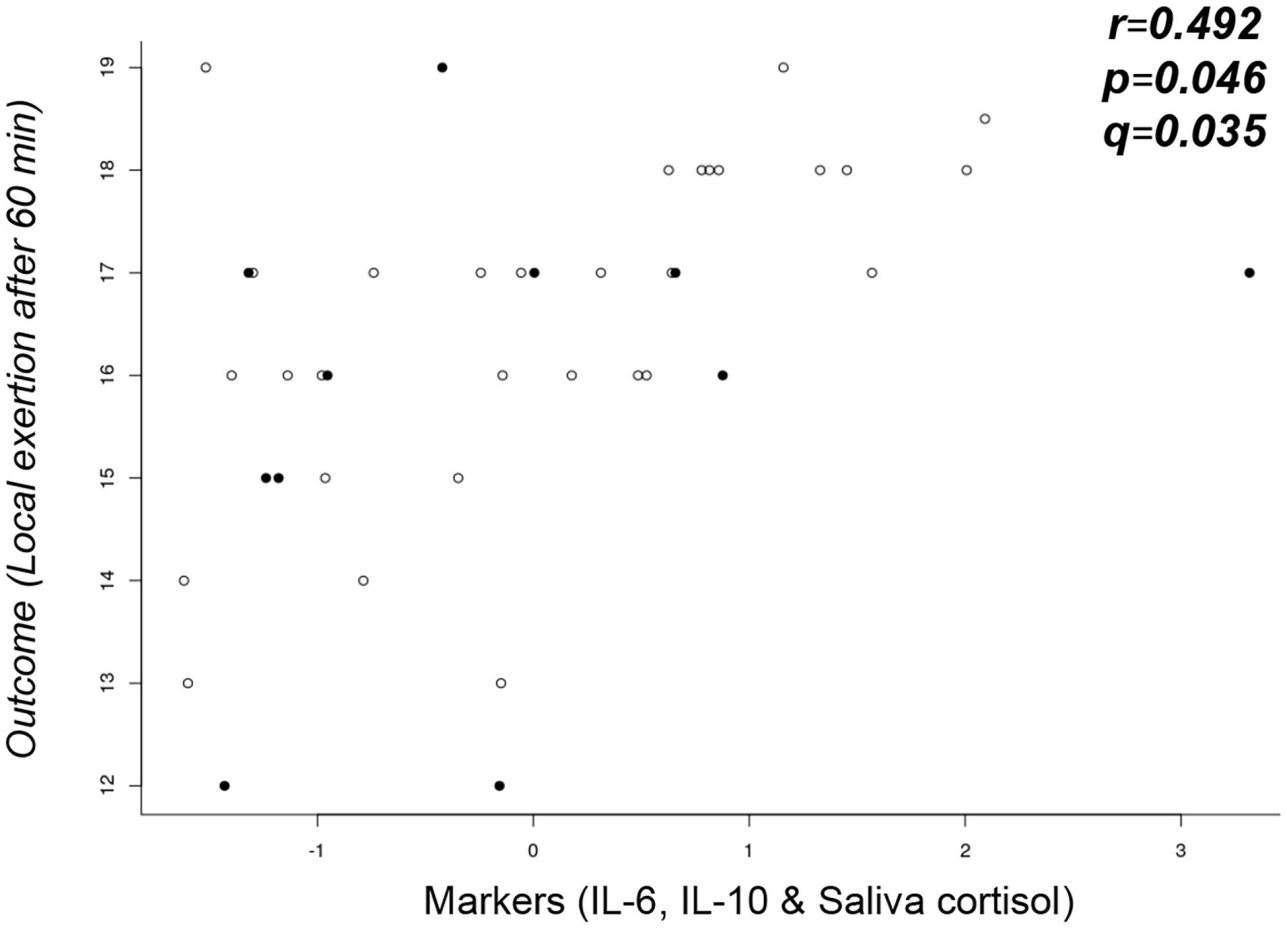
Significant correlation between the combination of IL‐6, IL‐10 and saliva cortisol with local exertion after 60 min. Black dots represent female participants and open dots represent male participants. Data were log2‐transformed and standardized prior to sparse Canonical Correlation analysis (sCCA) between biomarkers and symptoms and exertion after the exercise challenge. FDR estimates (*q*‐values) were calculated according to Storey (2002). IL—Interleukin.

No correlations were found between the L/R ratio and reported gastrointestinal symptoms after the exercise challenge and perceived exertion during and at the end of the challenge (Table S9). Using sparse canonical correlation analysis (sCCA), a significant correlation between the combination of IL6, IL10, and salivary cortisol with perceived local exertion after the exercise challenge was found (Table 3, Figure 4).

**TABLE 3:** Sparse canonical correlations between biomarkers and symptoms and perceived exertion.

Biomarkers	Coefficient	Outcome	Coefficient	sCCA
IL‐6	0.964	Local exertion after 60 min	1.000	*r* = 0.492 *p* = 0.046 *q* = 0.035
IL‐10	0.248			
Saliva cortisol	0.095			

*Note*: Sparse canonical correlations (sCCA) between biomarkers and symptoms and exertion. Data were log2‐transformed and standardized prior to sCCA analysis. FDR estimates (*q*‐values) were calculated according to Storey (2002).

Abbreviation: IL, Interleukin.

## 4 DISCUSSION

In this exploratory study, we investigated several markers of gut barrier and immune function after a high‐intensity exercise challenge to gain a better understanding of the acute systemic effects of an exercise‐induced increased intestinal permeability.

Following the exercise challenge, we observed a significant increase in urinary L/R ratio, a marker of small intestinal permeability. Additionally, we detected elevated plasma concentrations of I‐FABP. I‐FABP is a cytosolic protein primarily found at the tips of the small intestinal villi and released into the circulation upon enterocyte damage (Adriaanse et al., 2013; Bischoff et al., 2014; Grootjans et al., 2010; Guthmann et al., 2002; König et al., 2016; March et al., 2017; Pelsers et al., 2003; Thuijls et al., 2011). These findings are in line with numerous previous studies reporting increased L/R ratio and I‐FABP levels after different exercise challenges (Chantler et al., 2021; Costa et al., 2017; Engel et al., 2022; Lambert, 2004; March et al., 2017; Ogden et al., 2020; Smetanka et al., 1999; van Wijck, Lenaerts, Grootjans, et al., 2012; van Wijck, Lenaerts, Grootjans, et al., 2012).

As an increase in intestinal permeability may facilitate the translocation of bacteria from the external environment into the blood (Chantler et al., 2021; Costa et al., 2017), we assessed the number of bacterial 16S rRNA gene‐associated sequences and the concentrations of LPS in blood. We observed a significant increase in the number of microbial 16S rRNA gene‐associated sequences after exercise. However, the concentrations of LPS remained unaltered. A possible explanation of these seemingly opposed results could be that LPS is a challenging marker to analyze, as samples can be easily contaminated, among others (Citronberg et al., 2016). Additionally, LPS is a cell‐wall component of gram‐negative bacteria, while 16S rRNA is widely present in all bacteria. Moreover, an exercise‐induced increase in LPS concentrations seems to occur mostly due to exposure to exertional heat stress or ultra‐endurance exercise (Costa et al., 2019, 2020; Snipe et al., 2018).

An alternative to LPS could be the assessment of a targeted gut bacteria phylum, which might be less susceptible to external contamination. Hence, we analyzed the proportion of *Bacteroidota* DNA, a bacteria phylum common and highly abundant in the gastrointestinal tract (Rajilic‐Stojanovic & de Vos, 2014) to total bacterial DNA (*Bacteroidota*/total 16S rRNA ratio) as proposed by March et al., 2019 (March et al., 2019). The results of this analysis showed a (non‐significant) decrease of the *Bacteroidota*/total 16S rRNA ratio after the exercise challenge, suggesting that, although there was an increase in bacterial translocation into the bloodstream after the exercise challenge, the proportion of *Bacteroidota* that crossed the gastrointestinal barrier was less than the overall number of bacteria that may have crossed into the circulation.

Interestingly, in our study small intestinal permeability assessed by L/R ratio positively correlated with the *Bacteroidota*/total 16S rRNA ratio, even if L/R did not correlate with other markers of bacterial translocation (total 16S gene copy numbers and LPS). As markers of bacterial translocation are scarce, the use of *Bacteroidota*/total 16S rRNA ratio might be a promising alternative. However, a recent study found this ratio to be of poor analytical reliability and further research is needed before it can be established (Ogden et al., 2020). Moreover, even if a recent population study of almost 10,000 healthy humans suggests that the presence of microbes in the blood of healthy individuals is rare (Tan et al., 2023), it could still be a promising marker when investigating conditions where intestinal barrier function is challenged.

Even if the L/R ratio is an accurate and non‐invasive method to assess small intestinal permeability (Vanuytsel et al., 2021), it is rather burdensome for the participant and requires access to high‐performance liquid chromatography‐mass spectrometry (HPLC‐MS) instruments for the analysis of the sugars in urine (Vanuytsel et al., 2021). Hence, there is a need for surrogate markers. Previously, March et al. (2017) reported a moderate correlation between urinary L/R ratio and I‐FABP after a strenuous exercise protocol (20 min running at 80% of their VO_2_ max, *n* = 18 men), however, only at one out of two visits (March et al., 2017). The correlation in our male cohort at rest was also moderate but did not reach statistical significance. After exercise and when looking at the entire study population, we did not find such a correlation, similar to our previous study in healthy subjects that underwent a sauna dehydration challenge (Roca Rubio et al., 2021), suggesting that I‐FABP is not suitable as a direct surrogate marker for the L/R ratio. Instead, it probably reflects epithelial damage rather than intestinal permeability per se, and correlations between these two markers may vary depending on the context, i.e. the magnitude of the epithelial damage. In our laboratory‐based study, I‐FABP levels after the exercise challenged increased by an average of 400–500%, whereas in athletes that collapsed after a marathon race, levels were increased by over 1300% compared to baseline levels (Walter et al., 2021).

Exercise‐induced muscle damage promotes neutrophil translocation to the skeletal muscle and leads to elevated levels of ROS and oxidative stress, which could also play a role in exercise‐induced increase in intestinal permeability (Costa et al., 2017; Grootjans et al., 2016; Ji, 1999; Quindry et al., 2003; Scheffer & Latini, 2020; Van Wijck, Lenaerts, Van Bijnen, et al., 2012). The production of ROS during exercise also has positive effects, for example, improving antioxidative networking by uric acid production, which is the final product of purine metabolism and a major antioxidant in human blood (Ames et al., 1981; Kawamura & Muraoka, 2018; Mastaloudis et al., 2001). In animal models, uric acid appears to protect against oxidative stress after intestinal ischemia/reperfusion (Jiro Ogura et al., 2012) and ischemic neuronal injury (Yu et al., 1998; Zhang et al., 2017). In this study we observed an increase of uric acid concentrations after the exercise challenge which is in line with previous reports (Kawamura & Muraoka, 2018; Mastaloudis et al., 2001). Moreover, we found a moderate negative correlation between uric acid and L/R ratio, suggesting a possible protective effect of uric acid against small intestinal barrier damage.

In the male cohort, we observed a significant positive correlation between post‐exercise small intestinal permeability (L/R) and fecal chromogranin A levels. Chromogranin A is an acidic secretory protein found in vesicles of different secretory cells, such as in enteroendocrine cells, neurons, and immune cells of the gastrointestinal tract (Ohman et al., 2012; Sundin et al., 2018; Wagner et al., 2013). It has several different functions, including acting as a precursor for biologically active peptides and facilitating communication between intestinal immune cells, enteric neurons, and enteroendocrine cells (Ohman et al., 2012; Sundin et al., 2018). Among others, Chromogranin A has been negatively correlated with gut microbial diversity (Sundin et al., 2018; Zhernakova et al., 2016) and has been shown to be increased in irritable bowel syndrome (IBS) patients as well as to correlate with abdominal pain in IBS (Ohman et al., 2012). In mice, chromogranin A has also been shown to be involved in the paracellular regulation of the intestinal barrier (Muntjewerff et al., 2021). The variety of functions makes it difficult to hypothesize about the reason behind its correlation to the L/R ratio after exercise. However, it might be a potential surrogate marker to assess intestinal permeability instead of the L/R ratio, and this association should be further investigated in different cohorts and under different conditions. Nevertheless, it is important to consider that the correlation was observed in the male cohort after exercise only.

In our study, small intestinal permeability assessed with the L/R ratio did not correlate with any of the symptoms after the exercise challenge. While van Nieuwenhoven et al. (2004), reported that symptomatic athletes (*n* = 10) showed a higher increase of intestinal permeability due to strenuous exercise than athletes without gastrointestinal symptoms (*n* = 10) (van Nieuwenhoven et al., 2004), Karhu et al. (2017), reported an equal degree of exercise‐induced increased intestinal permeability in asymptomatic compared to symptomatic athletes (*n* = 17) (Karhu et al., 2017). An additional study by Pugh et al. also did not find a correlation between markers of intestinal barrier function and symptoms of gastrointestinal discomfort (*n* = 11) (Pugh et al., 2017). The authors argued that exercise tests in a laboratory environment often are of lower intensity and involve less mental stress compared to competitions, and thus might result in less gastrointestinal symptoms. Similarly, in our study, most participants only reported mild gastrointestinal symptoms, which could also explain that even using sCCA, a method that tries to find possible correlations by combining several outcomes, none of the biomarkers correlated with symptoms. Instead, applying sCCA, we found that perceived exertion correlated with the biomarker set of IL‐6, IL‐10 and salivary cortisol, suggesting that the inflammatory processes due to the strenuous exercise play an important role with regards to physical exhaustion.

One of the limitations of this study was the rather small sample size, especially with regards to the female cohort. However, this is a common limitation in studies with strenuous exercise challenges, and our study still comprises one of the larger sample sizes in this field of research. In addition, even if we did account for diurnal hormonal changes in our study by scheduling the biological sampling at the control and the challenge visit at the same time of the day, we did not consider potential hormonal fluctuation due to the menstrual cycle. Furthermore, we chose to analyze the correlations for the control and the exercise challenge separately, instead of using the delta values. We hypothesized that the correlations at rest might differ compared to the situation after the exercise challenge, with different physiological processes behind. In addition, as the effect of the exercise challenge was rather strong, we further hypothesized that subtracting the control values would not significantly impact the results.

It should further be noted that in this study all the participants were included based on their reported presence of gastrointestinal symptoms when training, even if they reported mostly mild symptoms after the exercise test. Nevertheless, this cohort may be more susceptible to the effects of ischemia in the gastrointestinal track than others. Moreover, this is a set of endurance‐trained subjects with a high VO_2_ max (females: 49.5 ± 4.6; males: 54.4 ± 4.9), and therefore the results and associations observed in this study should be interpreted with caution and verified in different cohorts.

## 5 CONCLUSIONS

The results obtained in this exploratory study show the impact of strenuous exercise on intestinal barrier function as well as other physiological markers. It seems that the barrier disruption as a result of the exercise challenge in this study was not sufficient to considerately impact gastrointestinal distress. We could not find a direct correlation between intestinal permeability and, for example, inflammatory markers which could suggest that other, not assessed, effects of strenuous exercise have a larger impact on these parameters. However, we found a negative correlation between L/R ratio and uric acid, which could point towards a potential positive effect of uric acid over the intestinal barrier. Furthermore, the results of this study propose further investigation of the *Bacteroidota*/total 16S rRNA ratio as well as fecal chromogranin A as potential surrogate markers for the L/R ratio to assess small intestinal permeability.

## FUNDING INFORMATION

The study was partially supported by the Knowledge Foundation Sweden (Grant reference number: 20110225) and Chr. Hansen A/S, Denmark.

## CONFLICT OF INTEREST STATEMENT

The authors declare no conflict of interest.

## ETHICS STATEMENT

The study was conducted according to the principles of the Declaration of Helsinki and its revisions, and ethical approval was obtained from the Central Ethical Review Board of Uppsala, Sweden (registration number 2015/077, amendment 2022‐02024‐02).

## CONSENT

All participants were recruited from the greater area of Örebro and gave their written informed consent before participation.

## Supporting information


Table S1.
Table S2.
Table S3.
Table S4.
Table S5.
Table S6.
Table S7.
Table S8.
Table S9.

## Data Availability

The datasets used and analyzed during the current study are available as a supplemental excel file.

## References

[phy216087-bib-0001] Adriaanse, M. P. M. , Tack, G. J. , Passos, V. L. , Damoiseaux, J. G. M. C. , Schreurs, M. W. J. , van Wijck, K. , Riedl, R. G. , Masclee, A. A. , Buurman, W. A. , Mulder, C. J. , & Vreugdenhil, A. C. E. (2013). Serum I‐FABP as marker for enterocyte damage in coeliac disease and its relation to villous atrophy and circulating autoantibodies. Alimentary Pharmacology & Therapeutics, 37(4), 482–490. 10.1111/apt.12194 23289539

[phy216087-bib-0002] Ames, B. N. , Cathcart, R. , Schwiers, E. , & Hochstein, P. (1981). Uric acid provides an antioxidant defense in humans against oxidant‐ and radical‐caused aging and cancer: A hypothesis. Proceedings of the National Academy of Sciences of the United States of America, 78(11), 6858–6862. 10.1073/pnas.78.11.6858 6947260 PMC349151

[phy216087-bib-0003] Billat, V. L. , Hill, D. W. , Pinoteau, J. , Petit, B. , & Koralsztein, J. P. (1996). Effect of protocol on determination of velocity at VO_2_ max and on its time to exhaustion. Archives of Physiology and Biochemistry, 104(3), 313–321. 10.1076/apab.104.3.313.12908 8793023

[phy216087-bib-0004] Bischoff, S. C. , Barbara, G. , Buurman, W. , Ockhuizen, T. , Schulzke, J. D. , Serino, M. , Tilg, H. , Watson, A. , & Wells, J. M. (2014). Intestinal permeability–A new target for disease prevention and therapy. BMC Gastroenterology, 14, 189. 10.1186/s12876-014-0189-7 25407511 PMC4253991

[phy216087-bib-0005] Borg, G. (1998). Borg's perceived exertion and pain scales. Human Kinetics.

[phy216087-bib-0006] Chantler, S. , Griffiths, A. , Matu, J. , Davison, G. , Jones, B. , & Deighton, K. (2021). The effects of exercise on indirect markers of gut damage and permeability: A systematic review and meta‐analysis. Sports Medicine, 51(1), 113–124. 10.1007/s40279-020-01348-y 33201454 PMC7806566

[phy216087-bib-0007] Citronberg, J. S. , Wilkens, L. R. , Lim, U. , Hullar, M. A. , White, E. , Newcomb, P. A. , Le Marchand, L. , & Lampe, J. W. (2016). Reliability of plasma lipopolysaccharide‐binding protein (LBP) from repeated measures in healthy adults. Cancer Causes & Control, 27(9), 1163–1166. 10.1007/s10552-016-0783-9 27392432 PMC5068910

[phy216087-bib-0008] Costa, R. J. S. , Camoes‐Costa, V. , Snipe, R. M. J. , Dixon, D. , Russo, I. , & Huschtscha, Z. (2019). Impact of exercise‐induced hypohydration on gastrointestinal integrity, function, symptoms, and systemic endotoxin and inflammatory profile. Journal of Applied Physiology (Bethesda, MD: 1985), 126(5), 1281–1291. 10.1152/japplphysiol.01032.2018 30896356

[phy216087-bib-0009] Costa, R. J. S. , Gaskell, S. K. , McCubbin, A. J. , & Snipe, R. M. J. (2020). Exertional‐heat stress‐associated gastrointestinal perturbations during Olympic sports: Management strategies for athletes preparing and competing in the 2020 Tokyo Olympic Games. Temperature (Austin), 7(1), 58–88. 10.1080/23328940.2019.1597676 32166105 PMC7053925

[phy216087-bib-0010] Costa, R. J. S. , Snipe, R. M. J. , Kitic, C. M. , & Gibson, P. R. (2017). Systematic review: Exercise‐induced gastrointestinal syndrome—Implications for health and intestinal disease. Alimentary Pharmacology & Therapeutics, 46(3), 246–265. 10.1111/apt.14157 28589631

[phy216087-bib-0011] Engel, S. , Mortensen, B. , Wellejus, A. , Vera‐Jimenez, N. , Struve, C. , Brummer, R. J. , Damholt, A. , Woods, T. , & Shanahan, F. (2022). Safety of Bifidobacterium breve, Bif195, employing a human exercise‐induced intestinal permeability model: A randomised, double‐blinded, placebo‐controlled, parallel group trial. Beneficial Microbes, 13(3), 243–252. 10.3920/BM2021.0173 35866597

[phy216087-bib-0012] Ganda Mall, J. P. , Fart, F. , Sabet, J. A. , Lindqvist, C. M. , Nestestog, R. , Hegge, F. T. , Keita, Å. V. , Brummer, R. J. , & Schoultz, I. (2020). Effects of dietary fibres on acute indomethacin‐induced intestinal hyperpermeability in the elderly: A randomised placebo controlled parallel clinical trial. Nutrients, 12(7), 1954. 10.3390/nu12071954 32629992 PMC7400264

[phy216087-bib-0013] Grootjans, J. , Lenaerts, K. , Buurman, W. A. , Dejong, C. H. C. , & Derikx, J. P. M. (2016). Life and death at the mucosal‐luminal interface: New perspectives on human intestinal ischemia‐reperfusion. World Journal of Gastroenterology, 22(9), 2760–2770. 10.3748/wjg.v22.i9.2760 26973414 PMC4777998

[phy216087-bib-0014] Grootjans, J. , Thuijls, G. , Verdam, F. , Derikx, J. P. , Lenaerts, K. , & Buurman, W. A. (2010). Non‐invasive assessment of barrier integrity and function of the human gut. World Journal of Gastrointestinal Surgery, 2(3), 61–69. 10.4240/wjgs.v2.i3.61 21160852 PMC2999221

[phy216087-bib-0015] Guthmann, F. , Borchers, T. , Wolfrum, C. , Wustrack, T. , Bartholomaus, S. , & Spener, F. (2002). Plasma concentration of intestinal‐ and liver‐FABP in neonates suffering from necrotizing enterocolitis and in healthy preterm neonates. Molecular and Cellular Biochemistry, 239(1–2), 227–234. 10.1023/A:1020508420058 12479590

[phy216087-bib-0016] Jeukendrup, A. E. , Vet‐Joop, K. , Sturk, A. , Stegen, J. H. J. C. , Senden, J. , Saris, W. H. M. , & Wagenmakers, A. J. M. (2000). Relationship between gastro‐intestinal complaints and endotoxaemia, cytokine release and the acute‐phase reaction during and after a long‐distance triathlon in highly trained men. Clinical Science (London, England), 98(1), 47–55. 10.1042/Cs19990258 10600658

[phy216087-bib-0017] Ji, L. L. (1999). Antioxidants and oxidative stress in exercise. Proceedings of the Society for Experimental Biology and Medicine, 222(3), 283–292. 10.1046/j.1525-1373.1999.d01-145.x 10601887

[phy216087-bib-0018] Jones, A. M. , & Doust, J. H. (1996). A 1% treadmill grade most accurately reflects the energetic cost of outdoor running. Journal of Sports Sciences, 14(4), 321–327. 10.1080/02640419608727717 8887211

[phy216087-bib-0019] Karhu, E. , Forsgard, R. A. , Alanko, L. , Alfthan, H. , Pussinen, P. , Hamalainen, E. , & Korpela, R. (2017). Exercise and gastrointestinal symptoms: Running‐induced changes in intestinal permeability and markers of gastrointestinal function in asymptomatic and symptomatic runners. European Journal of Applied Physiology, 117(12), 2519–2526. 10.1007/s00421-017-3739-1 29032392 PMC5694518

[phy216087-bib-0020] Kawamura, T. , & Muraoka, I. (2018). Exercise‐induced oxidative stress and the effects of antioxidant intake from a physiological viewpoint. Antioxidants (Basel), 7(9), 119. 10.3390/antiox7090119 30189660 PMC6162669

[phy216087-bib-0021] Keirns, B. H. , Koemel, N. A. , Sciarrillo, C. M. , Anderson, K. L. , & Emerson, S. R. (2020). Exercise and intestinal permeability: Another form of exercise‐induced hormesis? American Journal of Physiology. Gastrointestinal and Liver Physiology, 319(4), G512–G518. 10.1152/ajpgi.00232.2020 32845171

[phy216087-bib-0022] König, J. , Wells, J. , Cani, P. D. , Garcia‐Rodenas, C. L. , MacDonald, T. , Mercenier, A. , Whyte, J. , Troost, F. , & Brummer, R. J. (2016). Human intestinal barrier function in health and disease. Clinical and Translational Gastroenterology, 7(10), e196. 10.1038/ctg.2016.54 27763627 PMC5288588

[phy216087-bib-0023] Kulich, K. R. , Madisch, A. , Pacini, F. , Pique, J. M. , Regula, J. , Van Rensburg, C. J. , Ujszászy, L. , Carlsson, J. , Halling, K. , & Wiklund, I. K. (2008). Reliability and validity of the gastrointestinal symptom rating scale (GSRS) and quality of life in reflux and dyspepsia (QOLRAD) questionnaire in dyspepsia: A six‐country study. Health and Quality of Life Outcomes, 6, 12. 10.1186/1477-7525-6-12 18237386 PMC2276197

[phy216087-bib-0024] Lambert, G. P. (2004). Role of gastrointestinal permeability in exertional heatstroke. Exercise and Sport Sciences Reviews, 32(4), 185–190. 10.1097/00003677-200410000-00011 15604939

[phy216087-bib-0025] Lykkesfeldt, J. (2000). Determination of ascorbic acid and dehydroascorbic acid in biological samples by high‐performance liquid chromatography using subtraction methods: Reliable reduction with tris[2‐carboxyethyl]phosphine hydrochloride. Analytical Biochemistry, 282(1), 89–93. 10.1006/abio.2000.4592 10860503

[phy216087-bib-0026] March, D. S. , Jones, A. W. , Thatcher, R. , & Davison, G. (2019). The effect of bovine colostrum supplementation on intestinal injury and circulating intestinal bacterial DNA following exercise in the heat. European Journal of Nutrition, 58(4), 1441–1451. 10.1007/s00394-018-1670-9 29574607 PMC6561991

[phy216087-bib-0027] March, D. S. , Marchbank, T. , Playford, R. J. , Jones, A. W. , Thatcher, R. , & Davison, G. (2017). Intestinal fatty acid‐binding protein and gut permeability responses to exercise. European Journal of Applied Physiology, 117(5), 931–941. 10.1007/s00421-017-3582-4 28290057 PMC5388720

[phy216087-bib-0028] Mastaloudis, A. , Leonard, S. W. , & Traber, M. G. (2001). Oxidative stress in athletes during extreme endurance exercise. Free Radical Biology & Medicine, 31(7), 911–922. 10.1016/s0891-5849(01)00667-0 11585710

[phy216087-bib-0029] Muntjewerff, E. M. , Tang, K. , Lutter, L. , Christoffersson, G. , Nicolasen, M. J. T. , Gao, H. , Katkar, G. D. , Das, S. , Ter Beest, M. , Ying, W. , Ghosh, P. , El Aidy, S. , Oldenburg, B. , van den Bogaart, G. , & Mahata, S. K. (2021). Chromogranin A regulates gut permeability via the antagonistic actions of its proteolytic peptides. Acta Physiologica (Oxford, England), 232(2), e13655. 10.1111/apha.13655 33783968 PMC8341099

[phy216087-bib-0030] Ogden, H. B. , Fallowfield, J. L. , Child, R. B. , Davison, G. , Fleming, S. C. , Edinburgh, R. M. , Delves, S. K. , Millyard, A. , Westwood, C. S. , & Layden, J. D. (2020). Reliability of gastrointestinal barrier integrity and microbial translocation biomarkers at rest and following exertional heat stress. Physiological Reports, 8(5), e14374. 10.14814/phy2.14374 32170836 PMC7070100

[phy216087-bib-0031] Ogura, J. , Kuwayama, K. , Takaya, A. , Terada, Y. , Tsujimoto, T. , Koizumi, T. , Maruyama, H. , Fujikawa, A. , Takahashi, N. , Kobayashi, M. , Itagaki, S. , Hirano, T. , Yamaguchi, H. , & Iseki, K. (2012). Intestinal ischemia‐reperfusion increases efflux for uric acid via paracellular route in the intestine, but decreases that via transcellular route mediated by BCRP. Journal of Pharmacy & Pharmaceutical Sciences, 15(2), 294–304. 10.18433/j3w896 22579008

[phy216087-bib-0032] Ohman, L. , Stridsberg, M. , Isaksson, S. , Jerlstad, P. , & Simren, M. (2012). Altered levels of fecal chromogranins and secretogranins in IBS: Relevance for pathophysiology and symptoms? The American Journal of Gastroenterology, 107(3), 440–447. 10.1038/ajg.2011.458 22233694

[phy216087-bib-0033] Paisse, S. , Valle, C. , Servant, F. , Courtney, M. , Burcelin, R. , Amar, J. , & Lelouvier, B. (2016). Comprehensive description of blood microbiome from healthy donors assessed by 16S targeted metagenomic sequencing. Transfusion, 56(5), 1138–1147. 10.1111/trf.13477 26865079

[phy216087-bib-0034] Pelsers, M. M. A. L. , Namiot, Z. , Kisielewski, W. , Namiot, A. , Januszkiewicz, M. , Hermens, W. T. , & Glatz, J. F. C. (2003). Intestinal‐type and liver‐type fatty acid‐binding protein in the intestine. Tissue distribution and clinical utility. Clinical Biochemistry, 36(7), 529–535. 10.1016/S0009-9120(03)00096-1 14563446

[phy216087-bib-0035] Peters, H. P. F. , Bos, M. , Seebregts, L. , Akkermans, L. M. A. , Henegouwen, G. P. V. , Bol, E. , Mosterd, W. L. , & de Vries, W. R. (1999). Gastrointestinal symptoms in long‐distance runners, cyclists, and triathletes: Prevalence, medication, and etiology. American Journal of Gastroenterology, 94(6), 1570–1581. 10.1111/j.1572-0241.1999.01147.x 10364027

[phy216087-bib-0036] Pugh, J. N. , Impey, S. G. , Doran, D. A. , Fleming, S. C. , Morton, J. P. , & Close, G. L. (2017). Acute high‐intensity interval running increases markers of gastrointestinal damage and permeability but not gastrointestinal symptoms. Applied Physiology, Nutrition, and Metabolism, 42(9), 941–947. 10.1139/apnm-2016-0646 28511020

[phy216087-bib-0037] Pugh, J. N. , Sparks, A. S. , Doran, D. A. , Fleming, S. C. , Langan‐Evans, C. , Kirk, B. , Fearn, R. , Morton, J. P. , & Close, G. L. (2019). Four weeks of probiotic supplementation reduces GI symptoms during a marathon race. European Journal of Applied Physiology, 119(7), 1491–1501. 10.1007/s00421-019-04136-3 30982100 PMC6570661

[phy216087-bib-0038] Qamar, M. I. , & Read, A. E. (1987). Effects of exercise on mesenteric blood flow in man. Gut, 28(5), 583–587. Retrieved from https://www.ncbi.nlm.nih.gov/pubmed/3596339 http://gut.bmj.com/content/gutjnl/28/5/583.full.pdf 3596339 10.1136/gut.28.5.583 PMC1432887

[phy216087-bib-0039] Quindry, J. C. , Stone, W. L. , King, J. , & Broeder, C. E. (2003). The effects of acute exercise on neutrophils and plasma oxidative stress. Medicine and Science in Sports and Exercise, 35(7), 1139–1145. 10.1249/01.MSS.0000074568.82597.0B 12840634

[phy216087-bib-0040] Rajilic‐Stojanovic, M. , & de Vos, W. M. (2014). The first 1000 cultured species of the human gastrointestinal microbiota. FEMS Microbiology Reviews, 38(5), 996–1047. 10.1111/1574-6976.12075 24861948 PMC4262072

[phy216087-bib-0041] Roca Rubio, M. F. , Eriksson, U. , Brummer, R. J. , & Konig, J. (2021). Sauna dehydration as a new physiological challenge model for intestinal barrier function. Scientific Reports, 11(1), 15514. 10.1038/s41598-021-94814-0 34330970 PMC8324874

[phy216087-bib-0042] Rousu, J. , Agranoff, D. D. , Sodeinde, O. , Shawe‐Taylor, J. , & Fernandez‐Reyes, D. (2013). Biomarker discovery by sparse canonical correlation analysis of complex clinical phenotypes of tuberculosis and malaria. PLoS Computational Biology, 9(4), e1003018. 10.1371/journal.pcbi.1003018 23637585 PMC3630122

[phy216087-bib-0043] Scheffer, D. D. L. , & Latini, A. (2020). Exercise‐induced immune system response: Anti‐inflammatory status on peripheral and central organs. Biochimica et Biophysica Acta ‐ Molecular Basis of Disease, 1866(10), 165823. 10.1016/j.bbadis.2020.165823 32360589 PMC7188661

[phy216087-bib-0044] Schellekens, D. H. , Hundscheid, I. H. , Leenarts, C. A. , Grootjans, J. , Lenaerts, K. , Buurman, W. A. , Dejong, C. H. , & Derikx, J. P. (2017). Human small intestine is capable of restoring barrier function after short ischemic periods. World Journal of Gastroenterology, 23(48), 8452–8464. 10.3748/wjg.v23.i48.8452 29358855 PMC5752707

[phy216087-bib-0045] Smetanka, R. D. , Lambert, G. P. , Murray, R. , Eddy, D. , Horn, M. , & Gisolfi, C. V. (1999). Intestinal permeability in runners in the 1996 Chicago marathon. International Journal of Sport Nutrition, 9(4), 426–433. 10.1123/ijsn.9.4.426 10660873

[phy216087-bib-0046] Snipe, R. M. J. , Khoo, A. , Kitic, C. M. , Gibson, P. R. , & Costa, R. J. S. (2018). The impact of mild heat stress during prolonged running on gastrointestinal integrity, gastrointestinal symptoms, systemic endotoxin and cytokine profiles. International Journal of Sports Medicine, 39(4), 255–263. 10.1055/s-0043-122742 29415294

[phy216087-bib-0047] Storey, J. D. (2002). A direct approach to false discovery rates. Journal of the Royal Statistical Society, Series B: Statistical Methodology, 64(3), 479–498. 10.1111/1467-9868.00346

[phy216087-bib-0048] Sundin, J. , Stridsberg, M. , Tap, J. , Derrien, M. , Le Neve, B. , Dore, J. , Törnblom, H. , Simrén, M. , & Ohman, L. (2018). Fecal chromogranins and secretogranins are linked to the fecal and mucosal intestinal bacterial composition of IBS patients and healthy subjects. Scientific Reports, 8(1), 16821. 10.1038/s41598-018-35241-6 30429499 PMC6235916

[phy216087-bib-0049] Tan, C. C. S. , Ko, K. K. K. , Chen, H. , Liu, J. , Loh, M. , Consortium, S. G. K. H. , Chia, M. , & Nagarajan, N. (2023). No evidence for a common blood microbiome based on a population study of 9,770 healthy humans. Nature Microbiology, 8(5), 973–985. 10.1038/s41564-023-01350-w PMC10159858 36997797

[phy216087-bib-0050] ter Steege, R. W. , Geelkerken, R. H. , Huisman, A. B. , & Kolkman, J. J. (2012). Abdominal symptoms during physical exercise and the role of gastrointestinal ischaemia: A study in 12 symptomatic athletes. British Journal of Sports Medicine, 46(13), 931–935. 10.1136/bjsports-2011-090277 22021352

[phy216087-bib-0051] ter Steege, R. W. , Van der Palen, J. , & Kolkman, J. J. (2008). Prevalence of gastrointestinal complaints in runners competing in a long‐distance run: An internet‐based observational study in 1281 subjects. Scandinavian Journal of Gastroenterology, 43(12), 1477–1482. 10.1080/00365520802321170 18777440

[phy216087-bib-0052] Thuijls, G. , van Wijck, K. , Grootjans, J. , Derikx, J. P. , van Bijnen, A. A. , Heineman, E. , Dejong, C. H. , Buurman, W. A. , & Poeze, M. (2011). Early diagnosis of intestinal ischemia using urinary and plasma fatty acid binding proteins. Annals of Surgery, 253(2), 303–308. 10.1097/SLA.0b013e318207a767 21245670

[phy216087-bib-0053] van Nieuwenhoven, M. A. , Brouns, F. , & Brummer, R. J. (2004). Gastrointestinal profile of symptomatic athletes at rest and during physical exercise. European Journal of Applied Physiology, 91(4), 429–434. 10.1007/s00421-003-1007-z 14634826

[phy216087-bib-0054] van Wijck, K. , Lenaerts, K. , Grootjans, J. , Wijnands, K. A. , Poeze, M. , van Loon, L. J. , Dejong, C. H. , & Buurman, W. A. (2012). Physiology and pathophysiology of splanchnic hypoperfusion and intestinal injury during exercise: Strategies for evaluation and prevention. American Journal of Physiology. Gastrointestinal and Liver Physiology, 303(2), G155–G168. 10.1152/ajpgi.00066.2012 22517770

[phy216087-bib-0055] Van Wijck, K. , Lenaerts, K. , Van Bijnen, A. A. , Boonen, B. , Van Loon, L. J. C. , Dejong, C. H. C. , & Buurman, W. A. (2012). Aggravation of exercise‐induced intestinal injury by ibuprofen in athletes. Medicine and Science in Sports and Exercise, 44(12), 2257–2262. 10.1249/MSS.0b013e318265dd3d 22776871

[phy216087-bib-0056] van Wijck, K. , Lenaerts, K. , van Loon, L. J. , Peters, W. H. , Buurman, W. A. , & Dejong, C. H. (2011). Exercise‐induced splanchnic hypoperfusion results in gut dysfunction in healthy men. PLoS One, 6(7), e22366. 10.1371/journal.pone.0022366 21811592 PMC3141050

[phy216087-bib-0057] van Wijck, K. , van Eijk, H. M. H. , Buurman, W. A. , Dejong, C. H. C. , & Lenaerts, K. (2011). Novel analytical approach to a multi‐sugar whole gut permeability assay. Journal of Chromatography. B, Analytical Technologies in the Biomedical and Life Sciences, 879(26), 2794–2801. 10.1016/j.jchromb.2011.08.002 21862422

[phy216087-bib-0058] Vanuytsel, T. , Tack, J. , & Farre, R. (2021). The role of intestinal permeability in gastrointestinal disorders and current methods of evaluation. Frontiers in Nutrition, 8, 717925. 10.3389/fnut.2021.717925 34513903 PMC8427160

[phy216087-bib-0059] Wagner, M. , Stridsberg, M. , Peterson, C. G. , Sangfelt, P. , Lampinen, M. , & Carlson, M. (2013). Increased fecal levels of chromogranin A, chromogranin B, and secretoneurin in collagenous colitis. Inflammation, 36(4), 855–861. 10.1007/s10753-013-9612-4 23423580

[phy216087-bib-0060] Walter, E. , Gibson, O. R. , Stacey, M. , Hill, N. , Parsons, I. T. , & Woods, D. (2021). Changes in gastrointestinal cell integrity after marathon running and exercise‐associated collapse. European Journal of Applied Physiology, 121(4), 1179–1187. 10.1007/s00421-021-04603-w 33512586

[phy216087-bib-0061] Witten, D. M. , Tibshirani, R. , & Hastie, T. (2009). A penalized matrix decomposition, with applications to sparse principal components and canonical correlation analysis. Biostatistics, 10(3), 515–534. 10.1093/biostatistics/kxp008 19377034 PMC2697346

[phy216087-bib-0062] Yu, Z. F. , Bruce‐Keller, A. J. , Goodman, Y. , & Mattson, M. P. (1998). Uric acid protects neurons against excitotoxic and metabolic insults in cell culture, and against focal ischemic brain injury in vivo. Journal of Neuroscience Research, 53(5), 613–625. 10.1002/(SICI)1097-4547(19980901)53:5<613::AID-JNR11>3.0.CO;2-1 9726432

[phy216087-bib-0063] Zhang, B. , Yang, N. , Lin, S. P. , & Zhang, F. (2017). Suitable concentrations of uric acid can reduce cell death in models of OGD and cerebral ischemia‐reperfusion injury. Cellular and Molecular Neurobiology, 37(5), 931–939. 10.1007/s10571-016-0430-8 27709309 PMC11482132

[phy216087-bib-0064] Zhernakova, A. , Kurilshikov, A. , Bonder, M. J. , Tigchelaar, E. F. , Schirmer, M. , Vatanen, T. , Mujagic, Z. , Vila, A. V. , Falony, G. , Vieira‐Silva, S. , Wang, J. , Imhann, F. , Brandsma, E. , Jankipersadsing, S. A. , Joossens, M. , Cenit, M. C. , Deelen, P. , Swertz, M. A. , LifeLines cohort study , … Fu, J. (2016). Population‐based metagenomics analysis reveals markers for gut microbiome composition and diversity. Science, 352(6285), 565–569. 10.1126/science.aad3369 27126040 PMC5240844

[phy216087-bib-0065] Zuhl, M. N. , Lanphere, K. R. , Kravitz, L. , Mermier, C. M. , Schneider, S. , Dokladny, K. , & Moseley, P. L. (2014). Effects of oral glutamine supplementation on exercise‐induced gastrointestinal permeability and tight junction protein expression. Journal of Applied Physiology, 116(2), 183–191. 10.1152/japplphysiol.00646.2013 24285149 PMC3921361

