# Supplementary material for: Associations between various markers of intestinal barrier and immune function after a high‐intensity exercise challenge
Source: Physiol Rep. 2024 May 23;12(10):e16087. doi: 10.14814/phy2.16087 (PMC11116166; doi:10.14814/phy2.16087)
Supplement: Supplementary file 1 — Table S1. Table S2. Table S3. Table S4. Table S5. Table S6. Table S7. Table S8. Table S9. [file PHY2-12-e16087-s001.docx]

# *Supplemental material*

**Associations between various markers of intestinal barrier and immune function after a high-intensity exercise challenge**

Maria Fernanda Roca Rubio^1^, Mattias Folkesson^2^, Carolin Kremp^1^, Niklas Evertsson^1^, Dirk Repsilber^1^, Ulrika Eriksson^3^, John-Peter Ganda Mall^1^, Fawzi Kadi^2^, Robert J. Brummer^1^, Julia König^1*^

*^1^Nutrition-Gut-Brain Interactions Research Centre, School of Medical Sciences, Faculty of Medicine and Health, Örebro University, Örebro, Sweden. ^2^Division of Sports Sciences, School of Health Sciences, Faculty of Medicine and Health, Örebro University, Örebro, Sweden. ^3^Man‑Technology‑Environment (MTM) Research Centre, School of Science and Technology, Örebro University, Örebro, Sweden.*

*Corresponding author: Julia.Konig@oru.se

ORCIDs:

Maria Fernanda Roca Rubio: 0000-0002-4491-1776

Dirk Repsilber: 0000-0002-7173-5579

Ulrika Eriksson: 0000-0003-2392-0515

John-Peter Ganda Mall: 0000-0002-2120-7743

Fawzi Kadi: 0000-0002-9831-0896

Robert J. Brummer: 0000-0002-0362-0008

Julia König: 0000-0003-0466-1861

Supplemental Table 1: Gastrointestinal symptoms reported after exercise

|  | **Severity (reported by n (%))** | | | | | | | | |  |  |
| --- | --- | --- | --- | --- | --- | --- | --- | --- | --- | --- | --- |
|  | **None** | **Very mild** | **Mild** | | **Moderate** | | **Severe** | | **Total** | | |
|  | 0 | 1 | 2 | 3 | 4 | 5 | 6 | 7 |  | | |
| Belching | 20 (49%) | 4 (10%) | 5 (12%) | 8 (20%) | 2 (5%) | 1 (2.5%) | 1(2.5%) | - | **21 (51%)** | | |
| Heart burn | 37 (90%) | 1 (2.5%) | 1 (2.5%) | 1 (2.5%) | 1 (2.5%) | - | - | - | **4 (10%)** | | |
| Bloating | 27 (66%) | 2 (5%) | 7 (17%) | 2 (5%) | 3 (7%) | - | - | - | **14 (34%)** | | |
| Chest pain | 38 (93%) | 2 (5%) | - | - | - | 1 (2.5%) | - | - | **3 (7%)** | | |
| Nausea | 26 (63%) | 5 (12%) | 1 (2.5%) | 4 (10%) | 2 (5%) | 1 (2.5%) | 2 (5%) | - | **15 (36%)** | | |
| Vomit | 40 (97.5%) | - | - | 1 (2.5%) | - | - | - | - | **1 (2.5%)** | | |
| Abdominal pain | 25 (61%) | 3 (7%) | 3 (7%) | 3 (7%) | 6 (15%) | - | 1 (2.5%) | - | **16 (39%)** | | |
| Flatulence | 28 (68%) | 4 (10%) | 5 (12%) | 1 (2.5%) | 1 (2%) | 1 (2.5%) | 1 (2.5%) | - | **13 (32%)** | | |
| Urge to defaecate | 28 (68%) | 1 (2.5%) | 2 (5%) | 2 (5%) | 4 (10%) | 1 (2.5%) | 3 (7%) | - | **13 (32%)** | | |
| Diarrhoea | 39 (95%) | - | 1 (2.5%) | 1 (2.5%) | - | - | - | - | **2 (5%)** | | |
| Stitches | 19 (46%) | 3 (7%) | 6 (15%) | 3 (7%) | 8 (20%) | 1 (2.5%) | 1 (2.5%) | - | **22 (54%)** | | |
| Bloody diarrhoea | 41 (100%) | - | - | - | - | - | - | - | **0** | | |

**Supplemental Table 2. Sex differences among markers related to intestinal barrier function, inflammation, and stress**

|  | **Females (Median (IQR))** | | **p value** | **q value** | **Males (Median (IQR))** | | **p value** | **q value** | **Sex differences** | |
| --- | --- | --- | --- | --- | --- | --- | --- | --- | --- | --- |
|  | **Control** | **Post-exercise** |  |  | **Control** | **Post-exercise** |  |  | **Control** | **Post-exercise** |
| Urinary L/R | 0.023  (0.013-0.033) | 0.049  (0.036-0.074) | 0.005 | **0.013** | 0.033  (0.026-0.051) | 0.045  (0.032-0.062) | 0.001 | **0.002** | p=0.010  q=0.063 | p=0.359 |
| I-FABP (pg/ml) | 342  (204-437) | 1,819  (772-4,142) | 0.001 | **0.018** | 384  (240-529) | 1,778  (1,085-2,727) | <0.0001 | **0.002** | p=0.513 | p=0.828 |
| 16S rRNA (Copies 16S/ng DNA) | 54.4  (48.3-62.6) | 55.4  (48.7-71.2) | 0.064 |  | 49.3  (44.6-55.4) | 55.5  (50.8-62.4) | <0.0001 | **0.0009** | p=0.091 | p=0.890 |
| *Bacteroidota*/total 16S rRNA ratio | 0.0023  (0.0001-0.0833) | 0.0030  (0.0000-0.1378) | 0.589 |  | 0.0522  (0.0075-0.1058) | 0.0019  (0.0004-0.0627) | 0.012 | **0.022** | p=0.07 | p=0.921 |
| LPS (EU/ml) | 0.068  (0.055-0.096) | 0.075  (0.056-0.095) | 0.831 |  | 0.085  (0.056-0.134) | 0.088  (0.053-0.171) | 0.871 |  | p=0.552 | p=0.653 |
| FC (µg/ml) | 23  (10-41) | 23  (12-48) | 0.966 |  | 27  (21-52) | 28  (21-63) | 0.284 |  | p=0.441 | p=0.473 |
| IL-6 (pg/ml) | 0.82  (0.82-0.82) | 1.52  (0.82-4.82) | 0.031 | 0.069 | 0.82  (0.82-0.82) | 2.51  (0.825-4.649) | <0.0001 | **0.0006** | p=0.067 | p=0.312 |
| IL-8 (pg/ml) | 3.82  (3.21-5.59) | 6.42  (5.83-10.93) | 0.001 | **0.009** | 4.83  (3.28-5.83) | 7.16  (5.38-11.18) | <0.0001 | **0.0004** | p=0.783 | p=0.851 |
| IL-10 (pg/ml) | 0.82  (0.82-0.82) | 4.32  (2.30-4.76) | 0.001 | **0.006** | 0.822  (0.822-0.822) | 5.70  (3.21-11.62) | <0.0001 | **0.0004** | p=0.798 | p=0.249 |
| hs-CRP (mg/L) | 0.29  (0.16-1.15) | 0.30  (0.16-1.59) | 0.223 |  | 0.53  (0.37-0.99) | 0.60  (0.36-1.14) | 0.883 |  | p=0.141 | p=0.107 |
| MPO (ng/ml) | 26.4  (16.6-31.6) | 36.8  (26.4-38.5) | 0.002 | **0.009** | 26.9  (22.9-31.5) | 38.6  (31.8-49.4) | <0.0001 | **0.0003** | p=0.404 | p=0.141 |
| PC (U/ml) | 156.6  (134.4-195.8) | 157.8  (142.4 190.6) | 0.831 |  | 162.3  (136.9-203.6) | 185.9  (148.3-220.1) | 0.029 | **0.047** | p=0.612 | p=0.156 |

|  |  | | **p value** | **q value** |  | | **p value** | **q value** |  | |
| --- | --- | --- | --- | --- | --- | --- | --- | --- | --- | --- |
|  | **Females (Median (IQR))** | |  |  | **Males (Median (IQR))** | |  |  | **Sex differences** | |
|  | **Control** | **Post-exercise** |  |  | **Control** | **Post-exercise** |  |  | **Control** | **Post-exercise** |
| Uric Acid (µmol/L) | 146.2  (138.5-200.7) | 213.7  (200.1-223.0) | 0.002 | **0.007** | 260.2  (218.5-282.7) | 293.4  (255.2-313.6) | <0.0001 | **0.0003** | p=<0.0001  **q=0.002** | p=0.0001  **q=0.002** |
| HBD2 (ng/ml) | 31.0  (13.2-52.3) | 29.9  (14.2-72.5) | 0.695 |  | 64.5  (41.4-157.8) | 54.5  (32.3-117.3) | 0.859 |  | p=0.006  q=0.054 | p=0.078 |
| CgA (nmol/g) | 0.230  (0.140-0.510) | 0.260  (0.160-0.770) | 0.437 |  | 0.195  (0.147-0.567) | 0.255  (0.167-0.750) | 0.048 | 0.082 | p=0.936 | p=0.936 |
| CK (µkat/L) | 1.4  (0.9-4.0) | 1.7  (1.3-3.1) | 0.751 |  | 2.0  (1.4-3.5) | 3.3  (2.1-4.5) | 0.031 | **0.046** | p=0.348 | p=0.006  q=0.054 |
| sIgA (µg/ml) | 104.3  (74.4-149.7) | 87.9  (81.1-163.0) | 0.966 |  | 111.2  (86.9-179.2) | 125.7  (99.5-190.5) | 0.109 |  | p=0.374 | p=0.183 |
| Saliva cortisol (µg/dl) | 0.307  (0.199-0.380) | 1.158  (0.751-1.437) | 0.002 | **0.006** | 0.256  (0.158-0.359) | 1.079  (0.800-1.260) | <0.0001 | **0.0002** | p=0.129 | p=0.344 |

Median and interquartile ranges (IQR) are shown. Urinary L/R – lactulose/rhamnose excretion ratio. I‑FABP – intestinal fatty acid-binding protein. 16S rRNA – 16S ribosomal RNA gene copy numbers. LPS – lipopolysaccharides. FC – faecal calprotectin. IL – Interleukin**.** hs-CRP – high sensitivity C-reactive protein. MPO – myeloperoxidase. PC - protein carbonyls. HBD2 – faecal human ß-defensin 2. CgA – faecal chromogranin A. CK - creatine kinase. sIgA – salivary soluble immunoglobulin A. Difference between the two conditions was assessed by paired Wilcoxon signed-rank test. Sex differences were assessed by the Mann-Whitney U test. The Benjamin-Hochberg procedure with a false discovery rate FDR of <5%; was used to corrected for multiple comparisons. q-values smaller than 0.05 indicate significant differences and are highlighted in bold.

**Supplemental Table 3: Correlations among markers of barrier function at control visit**

|  |  | **I-FABP** | **16S rRNA** | ***Bacteroidota*/ total 16S rRNA** | **LPS** |
| --- | --- | --- | --- | --- | --- |
| ***L/R*** | *All* | r=0.239  p=0.132 | r=-0.333  p=0.034  q=0.427 | r=0.184  p=0.249 | r=-0.019  p=0.908 |
|  | *Females* | r=0.027  p=0.946 | r=-0.382  p=0.248 | r=0.091  p=0.796 | r=0.082  p=0.818 |
|  | *Males* | r=0.447  p=0.013  q=0.677 | r=-0.254  p=0.175 | r=0.179  p=0.343 | r=-0.051  p=0.791 |
|  | ***I-FABP*** | *All* | r=-0.154  p=0.337 | r=0.084  p=0.603 | r=0.135  p=0.401 |
|  |  | *Females* | r=0.318  p=0.342 | r=-0.109  p=0.755 | r=0.182  p=0.595 |
|  |  | *Males* | r=-0.175  p=0.356 | r=-0.009  p=0.964 | r=0.064  p=0.736 |
|  |  | ***16S rRNA*** | *All* | r=-0.080  p=0.618 | r=-0.270  p=0.088 |
|  |  |  | *Females* | r=-0.309  p=0.356 | r=-0.309  p=0.356 |
|  |  |  | *Males* | r=0.106  p=0.578 | r=-0.235  p=0.211 |
|  |  |  | ***Bacteroidota*/ total 16S rRNA** | *All* | r=0.095  p=0.556 |
|  |  |  |  | *Females* | r=0.464  p=0.155 |
|  |  |  |  | *Males* | r=-0.033  p=0.861 |

Correlations were performed by a two-tailed Spearman’s correlation analysis and corrected for multiplicity by the Benjamin-Hochberg procedure. p-values smaller than 0.05 and q-values smaller than 0.05 were considered statistically significant. L/R – urinary lactulose/rhamnose excretion ratio. I‑FABP – intestinal fatty acid-binding protein. 16S rRNA – 16S ribosomal RNA gene copy numbers. LPS – lipopolysaccharides.

**Supplemental Table 4. Correlations among markers of barrier function after exercise**

|  |  | **I-FABP** | **16S rRNA** | ***Bacteroidota*/ total 16S rRNA** | **LPS** |
| --- | --- | --- | --- | --- | --- |
| ***L/R*** | *All* | r=0.283  p=0.073 | r=-0.369  p=0.019  q=0.139 | **r=0.517**  **p=0.0006**  **q=0.031** | r=0.175  p=0.273 |
|  | *Females* | r=0.300  p=0.371 | r=-0.442  p=0.204 | r=0.791  p=0.009  q=0.092 | r=0.400  p=0.225 |
|  | *Males* | r=0.230  p=0.221 | r=-0.327  p=0.078 | r=0.367  p=0.046  q=0.466 | r=0.125  p=0.511 |
|  | ***I-FABP*** | *All* | r=-0.095  p=0.559 | r=0.151  p=0.352 | r=0.112  p=0.487 |
|  |  | *Females* | r=0.212  p=0.560 | r=0.325  p=0.360 | r=0.618  p=0.048  q= 1.218 |
|  |  | *Males* | r=-0.164  p=0.388 | r=0.009  p=0.962 | r=-0.048  p=0.802 |
|  |  | ***16S rRNA*** | *All* | r=-0.314  p=0.048  q=0.411 | r=-0.270  p=0.088 |
|  |  |  | *Females* | r=-0.411  p=0.240 | r=-0.309  p=0.356 |
|  |  |  | *Males* | r=-0.270  p=0.149 | r=-0.235  p=0.211 |
|  |  |  | ***Bacteroidota*/ total 16S rRNA** | *All* | r=0.166  p=0.307 |
|  |  |  |  | *Females* | r=0.239  p=0.508 |
|  |  |  |  | *Males* | r=0.103  p=0.586 |

Correlations were performed by a two-tailed Spearman’s correlation analysis and corrected for multiplicity by the Benjamin-Hochberg procedure. p-values smaller than 0.05 and q-values smaller than 0.05 were considered statistically significant and are highlighted in bold. L/R – urinary lactulose/rhamnose excretion ratio. I‑FABP – intestinal fatty acid-binding protein. 16S rRNA – 16S ribosomal RNA gene copy numbers. LPS – lipopolysaccharides.

**Supplemental Table 5. Correlations among markers of barrier function and markers of inflammatory immune response and oxidative stress at the control visit**

|  |  | **FC** | **IL-6** | **IL-8** | **IL-10** | **hs-CRP** | **MPO** | **PC** | **Uric acid** |
| --- | --- | --- | --- | --- | --- | --- | --- | --- | --- |
| **L/R** | *All* | r=0.124 p=0.439 | r=-0.081 p=0.616 | r=0.100 p=0.535 | r=0.085 p=0.596 | r=0.063 p=0.697 | r=0.126 p=0.434 | r=0.265 p=0.094 | r=0.010 p=0.949 |
|  | *Females* | r=0.445 p=0.173 | r=-0.094 p=0.800 | r=-0.182 p=0.595 | r=0.300 p=0.545 | r=0.018 p=0.967 | r=0.045 p=0.903 | r=0.436 p=0.183 | r=0.273 p=0.418 |
|  | *Males* | r=-0.059 p=0.755 | r=0.054 p=0.778 | r=0.192 p=0.309 | r=0.032 p=0.866 | r=0.045 p=0.815 | r=0.070 p=0.715 | r=0.144 p=0.550 | r=-0.444 p=0.014 q=0.357 |
| **I-FABP** | *All* | r=0.028 p=0.863 | r=0.041 p=0.801 | r=0.005 p=0.976 | r=0.276 p=0.080 | r=0.242 p=0.128 | r=0.054 p=0.737 | r=-0.186 p=0.244 | r=-0.115 p=0.473 |
|  | *Females* | r=-0.073 p=0.838 | r=0.054 p=0.891 | r=0.000 p=1.000 | r=0.400 p=0.364 | r=0.396 p=0.227 | r=-0.064 p=0.860 | r=-0.464 p=0.155 | r=0.109 p=0.755 |
|  | *Males* | r=0.103 p=0.590 | r=0.139 p=0.462 | r=0.033 p=0.864 | r=0.268 p=0.152 | r=0.203 p=0.282 | r=0.067 p=0.727 | r=-0.097 p=0.609 | r=-0.337 p=0.068 |
| **16S rRNA** | *All* | r=0.030 p=0.852 | r=0266 p=0.093 | r=0.102 p=0.527 | r=0.324 p=0.039 q=0.932 | r=0.202 p=0.206 | r=-0.118 p=0.463 | r=-0.203 p=0.202 | r=-0.153 p=0.340 |
|  | *Females* | r=0.364 p=0.273 | r=0.512 p=0.127 | r=0.355 p=0.286 | r=0.300 p=0.545 | r=0.009 p=0.989 | r=0.009 p=0.989 | r=-0.136 p=0.694 | r=-0.200 p=0.557 |
|  | *Males* | r=-0.026 p=0.890 | r=0.075 p=0.693 | r=0.051 p=0.789 | r=0.311 p=0.094 | r=0.329 p=0.076 | r=-0.088 p=0.645 | r=-0.228 p=0.226 | r=0.126 p=0.507 |
| ***Bacteroidota*/ total 16S rRNA** | All | r=-0.085 p=0.598 | r=0.042 p=0.795 | r=0.207 p=0.194 | r=0.183 p=0.251 | r=-0.149 p=0.352 | r=0.048 p=0.766 | r=0.115 p=0.475 | r=0.198 p=0.214 |
|  | *Females* | r=0.064 p=0.860 | r=0.189 p=0.600 | r=0.091 p=0.796 | r=0.400 p=0.364 | r=-0.239 p=0.481 | r=-0.445 p=0.173 | r=0.027 p=0.946 | r=0.382 p=0.248 |
|  | *Males* | r=-0171 p=0.336 | r=0.075 p=0.693 | r=0.216  p=0.251 | r=0.054 p=0.778 | r=-0.110 p=0.565 | r=0.164 p=0.387 | r=0.154 p=0.417 | r=-0.066 p=0.730 |
| **LPS** | *All* | r=0.132 p=0.409 | r=0.003 p=0.987 | r=0.032 p=0.844 | r=-0.109 p=0.497 | r=0.063 p=0.697 | r=0.039 p=0.809 | r=-0.188 p=0.240 | r=0.132 p=0.410 |
|  | *Females* | r=-0.100 p=0.776 | r=0.243 p=0.491 | r=0.445 p=0.173 | r=0.300 p=0.545 | r=0.018 p=0.967 | r=-0.855 p=0.001 q=0.074 | r=0.000 p=1.000 | r=-0.018 p=0.967 |
|  | *Males* | r=-0.250 p=0.276 | r=-0.032 p=0.866 | r=-0.089 p=0.641 | r=-0.268 p=0.152 | r=0.045 p=0.815 | r=0.354 p=0.055 | r=-0.250 p=0.183 | r=0.103 p=0.586 |

Correlations were performed by a two-tailed Spearman’s correlation analysis and corrected for multiplicity by the Benjamin-Hochberg procedure. p-values smaller than 0.05 and q-values smaller than 0.05 were considered statistically significant. L/R – urinary lactulose/rhamnose excretion ratio. I‑FABP – intestinal fatty acid-binding protein. 16S rRNA – 16S ribosomal RNA gene copy numbers. LPS – lipopolysaccharides. IL – interleukin. hs‑CRP – high sensitivity C-reactive protein. MPO – myeloperoxidase. PC – protein carbonyls.

**Supplemental Table 6. Correlations among biomarkers of barrier function and biomarkers of inflammatory immune response and oxidative stress after exercise**

|  |  | **FC** | **IL-6** | **IL-8** | **IL-10** | **hs-CRP** | **MPO** | **PC** | **Uric acid** |
| --- | --- | --- | --- | --- | --- | --- | --- | --- | --- |
| **L/R** | *All* | r=0.195 p=0.227 | r=0.089 p=0.580 | r=0.028  p=0.863 | r=-0.074 p=0.646 | r=-0.213  p=0.182 | r=-0.138  p=0.390 | r=-0.063  p=0.698 | **r=-0.480 p=0.002 q=0.048** |
|  | *Females* | r=0.109 p=0.755 | r=0.105 p=0.764 | r=0.045  p=0.903 | r=-0.400 p=0.225 | r=-0.147  p=0.670 | r=-0.527  p=0.100 | r=-0.182  p=0.595 | r=-0.115 p=0.759 |
|  | *Males* | r=0.264 p=0.166 | r=0.106 p=0.578 | r=0.042  p=0.824 | r=0.002 p=0.993 | r=-0.238  p=0.206 | r=0.021  p=0.912 | r=0.003  p=0.986 | r=-0.418 p=0.024 q=0.191 |
| **I-FABP** | *All* | r=0.165 p=0.308 | r=0.220 p=0.168 | r=0.057  p=0.722 | r=0.119  p=0.459 | r=-0.052  p=0.745 | r=-0.094  p=0.557 | r=-0.086  p=0.595 | r=-0.210  p=0.200 |
|  | *Females* | r=0.309 p=0.356 | r=0.629 p=0.044 q= 2.245 | r=0.500  p=0.122 | r=0.109  p=0.755 | r=0.028  p=0.945 | r=-0.245  p=0.468 | r=-0.227  p=0.503 | r=0.152  p=0.682 |
|  | *Males* | r=0.092 p=0.636 | r=-0.007 p=0.969 | r=-0.112  p=0.554 | r=0.053 p=0.782 | r=-0.125  p=0.510 | r=0.005  p=0.980 | r=-0.014  p=0.943 | r=-0.269  p=0.157 |
| **16S rRNA** | *All* | r=0.392 p=0.014 q=0.348 | r=0.210 p=0.192 | r=0.002  p=0.991 | r=0.055 p=0.735 | r=0.154  p=0.342 | r=-0.004  p=0.978 | r=-0.054  p=0.742 | r=0.376 p=0.020 q=0.257 |
|  | *Females* | r=0.661 p=0.044 q=0.372 | r=0.537 p=0.116 | r=0.394  p=0.263 | r=0.600  p=0.073 | r=0.141  p=0.703 | r=0.067  p=0.865 | r=0.479  p=0.166 | r=-0.250  p=0.521 |
|  | *Males* | r=0.292 p=0.124 | r=0.074 p=0.697 | r=-0.109 p=0.567 | r=-0.164 p=0.385 | r=0.321  p=0.083 | r=-0.080  p=0.675 | r=-0.167  p=0.378 | r=0.553  p=0.002  q=0.095 |
| ***Bacteroidota*/ total 16S rRNA** | All | r=-0.005 p=0.976 | r=0.144 p=0.376 | r=0.223 p=0.166 | r=0.199  p=0.217 | r=-0.378 p=0.016 q=0.277 | r=-0.014  p=0.933 | r=-0.026  p=0.876 | r=-0.169  p=0.309 |
|  | *Females* | r=0.080 p=0.835 | r=0.111 p=0.766 | r=0.178  p=0.627 | r=-0.067  p=0.862 | r=-0.453  p=0.191 | r=-0.472  p=0.172 | r=-0.387  p=0.271 | r=0.444  p=0.233 |
|  | *Males* | r=-0.086 p=0.656 | r=0.153 p=0.418 | r=0.249  p=0.185 | r=0.300 p=0.107 | r=-0.309 p=0.096 | r=0.199  p=0.291 | r=0.085  p=0.654 | r=-0.145 p=0.453 |
| **LPS** | *All* | r=0.123 p=0.448 | r=0.053 p=0.744 | r=0.245  p=0.122 | r=-0.135  p=0.399 | r=0.074 p=0.646 | r=0.026  p=0.870 | r=-0.032  p=0.843 | r=0.890  p=0.590 |
|  | *Females* | r=-0.136 p=0.694 | r=0.267 p=0.427 | r=0.682 p=0.025 q=1.282 | r=-0.327 p=0.327 | r=-0.339  p=0.307 | r=-0.564  p=0.076 | r=0.118  p=0.735 | r=-0.045  p=0.918 |
|  | *Males* | r=0.204 p=0.287 | r=-0.026 p=0.891 | r=0.185 p=0.327 | r=-0.124 p=0.513 | r=0.179  p=0.345 | r=0.179  p=0.345 | r=-0.051  p=0.787 | r=0.161  p=0.404 |

Correlations were performed by a two-tailed Spearman’s correlation analysis and corrected for multiplicity by the Benjamin-Hochberg procedure. p-values smaller than 0.05 and q-values smaller than 0.05 were considered statistically significant and are highlighted in bold. L/R – urinary lactulose/rhamnose excretion ratio. I‑FABP – intestinal fatty acid-binding protein. 16S rRNA – 16S ribosomal RNA gene copy numbers. LPS – lipopolysaccharides. IL – interleukin. hs‑CRP – high sensitivity C-reactive protein. MPO – myeloperoxidase. PC – protein carbonyls.

**Supplemental Table 7. Correlations among markers of intestinal barrier function and exploratory markers at the control visit**

|  |  | **HBD2** | **CgA** | **CK** | **sIgA** | **Saliva cortisol** |
| --- | --- | --- | --- | --- | --- | --- |
| **L/R** | *All* | r=0.011  p=0.949 | r=0.182  p=0.254 | r=-0.058  p=0.716 | r=0.091  p=0.570 | r=-0.213  p=0.182 |
|  | *Females* | r=-0.236  p=0.513 | r=0.265  p=0.428 | r=-0.115  p=0.647 | r=-0.727  p=0.014  q=0.245 | r=-0.147  p=0. 670 |
|  | *Males* | r=-0164  p=0.413 | r=0.148  p=0.435 | r=-0.034  p=0.857 | r=0.299  p=0.108 | r=-0.238  p=0.206 |
| **I-FABP** | *All* | r=-0.106  p=0.532 | r=0.018  p=0.913 | r=0.242  p=0.128 | r=0.232  p=0.144 | r=-0.052  p=0.745 |
|  | *Females* | r=0.006  p=1.000 | r=-0.201  p=0.551 | r=0.396  p=0.227 | r=0.373  p=0.261 | r=0.028  p=0.945 |
|  | *Males* | r=-0.142  p=0.481 | r=0.079  p=0.679 | r=0.203  p=0.282 | r=0.196  p=0.300 | r=-0.125  p=0.510 |
| **16S rRNA** | *All* | r=-0.065  p=0.700 | r=-0.176  p=0.271 | r=0.033  p=0.837 | r=0.058  p=0.719 | r=0.154  p=0.342 |
|  | *Females* | r=0.442  p=0.204 | r=-0.283  p=0.396 | r=0.032  p=0.930 | r=0.527  p=0.100 | r=0.141  p=0.703 |
|  | *Males* | r=-0.047  p=0.816 | r=-0.162  p=0.391 | r=0.061  p=0.746 | r=0.028  p=0.884 | r=0.321  p=0.083 |
| ***Bacteroidota*/ total 16S rRNA** | All | r=0.098  p=0.564 | r=0.040  p=0.803 | r=0.089  p=0.581 | r=-0.011  p=0.946 | r=0.015  p=0.925 |
|  | *Females* | r=-0212.  p=0.560 | r=0.055  p=0.876 | r=-0.137  p=0.687 | r=-0.136  p=0.560 | r=0.364  p=0.273 |
|  | *Males* | r=0.145  p=0.470 | r=-0.033  p=0.861 | r=0.192  p=0.308 | r=-0.074  p=0.696 | r=0.022  p=0.908 |
| **LPS** | *All* | r=0.123  p=0.468 | r=0.199  p=0.213 | r=0.157  p=0.328 | r=-0.112  p=0.486 | r=0.074  p=0.646 |
|  | *Females* | r=-0.006  p=1.000 | r=-0.224  p=0.505 | r=0.178  p=0.599 | r=0.064  p=0.860 | r=-0.339  p=0.307 |
|  | *Males* | r=0.093  p=0.643 | r=0.155  p=0.413 | r=0.117  p=0.538 | r=-0.185  p=0.328 | r=0.179  p=0.345 |

Correlations were performed by a two-tailed Spearman’s correlation analysis and corrected for multiplicity by the Benjamin-Hochberg procedure. p-values smaller than 0.05 and q-values smaller than 0.05 were considered statistically significant. L/R – urinary lactulose/rhamnose excretion ratio. I‑FABP – intestinal fatty acid-binding protein. 16S rRNA – 16S ribosomal RNA gene copy number. LPS – lipopolysaccharides. HBD2 – faecal human ß-defensin 2. CgA – faecal chromogranin A. CK – creatine kinase. sIgA – salivary soluble immunoglobulin A.

**Supplemental Table 8. Correlations among markers of intestinal barrier function and exploratory markers after exercise**

|  |  | **HBD2** | **CgA** | **CK** | **sIgA** | **Saliva cortisol** |
| --- | --- | --- | --- | --- | --- | --- |
| **L/R** | *All* | r=-0.137  p=0.419 | r=0.396  p=0.010  q=0.089 | r=-0.222  p=0.163 | r=-0.022  p=0.891 | r=0.292  p=0.064 |
|  | *Females* | r=0.115  p=0.759 | r=-0.023  p=0.952 | r=-0.371  p=0.260 | r=-0.236  p=0.485 | r=-0.327  p=0.327 |
|  | *Males* | r=-0.192  p=0.337 | **r=0.555**  **p=0.001**  **q=0.037** | r=-0.041  p=0.828 | r=0.083  p=0.661 | r=0.498  p=0.005  q=0.065 |
| **I-FABP** | *All* | r=-0.289  p=0.083 | r=0.040  p=0.806 | r=0.079  p=0.623 | r=0.028  p=0.861 | r=0.004  p=0.980 |
|  | *Females* | r=-0.212  p=0.560 | r=-0.351  p=0.289 | r=0.146  p=0.666 | r=0.591  p=0.061 | r=0.055  p=0.881 |
|  | *Males* | r=-0.281  p=0.156 | r=0.196  p=0.301 | r=0.190  p=0.315 | r=-0.224  p=0.233 | r=-0.038  p=0.844 |
| **16S rRNA** | *All* | r=0.048  p=0.782 | r=-0.101  p=0.536 | r=0.175  p=0.279 | r=0.141  p=0.386 | r=0.113  p=0.488 |
|  | *Females* | r=-0.267  p=0.493 | r=0.079  p=0.830 | r=0.147  p=0.685 | r=0.370  p=0.296 | r=0.685  p=0.035  q=0.333 |
|  | *Males* | r=0.226  p=0.257 | r=-0.158  p=0.403 | r=0.198  p=0.294 | r=0.065  p=0.734 | r=-0.081  p=0.670 |
| ***Bacteroidota*/ total 16S rRNA** | All | r=0.045  p=0.794 | r=0.159  p=0.326 | r=-0.159  p=0.326 | r=0.008  p=0.959 | r=0.096  p=0.554 |
|  | *Females* | r=0.268  p=0.481 | r=-0.474  p=0.168 | r=-0.223  p=0.532 | r=-0.104  p=0.781 | r=-0.288  p=0.420 |
|  | *Males* | r=-0.015  p=0.940 | r=0.408  p=0.025  q=0.319 | r=-0.014  p=0.942 | r=0.030  p=0.875 | r=0.274  p=0.144 |
| **LPS** | *All* | r=0.056  p=0.742 | r=0.139  p=0.386 | r=-0.128  p=0.425 | r=0.296  p=0.060 | r=0.224  p=0.160 |
|  | *Females* | r=-0.067  p=0.865 | r=-0.205  p=0.543 | r=-0.229  p=0.495 | r=0.427  p=0.193 | r=-0.391  p=0.237 |
|  | *Males* | r=0.082  p=0.683 | r=-0.184  p=0.331 | r=-0.133  p=0.484 | r=0.174  p=0.358 | r=0.376  p=0.040  q=0.132 |

Correlations were performed by a two-tailed Spearman’s correlation analysis and corrected for multiplicity by the Benjamin-Hochberg procedure. p-values smaller than 0.05 and q-values smaller than 0.05 were considered statistically significant and are highlighted in bold. L/R – urinary lactulose/rhamnose excretion ratio. I‑FABP – intestinal fatty acid-binding protein. 16S rRNA – 16S ribosomal RNA gene copy number. LPS – lipopolysaccharide. HBD2 –faecal human ß-defensin 2. CgA – faecal chromogranin A. CK – creatine kinase. sIgA – salivary soluble immunoglobulin A.

**Supplemental Table 9. Correlations among L/R ratio and gastrointestinal symptoms and exertion after exercise**

|  | **L/R ratio** | | |
| --- | --- | --- | --- |
|  | *All* | *Females* | *Males* |
| **Blenching** | r=0.151  p=0.360 | r=0.543  p=0.093 | r=0.129  p=0.513 |
| **Heartburn** | r=-0.282  p=0.082 | - | r=-0.298  p=0.123 |
| **Bloating** | r=0.055  p=0.739 | r=0.132  p=0.701 | r=0.042  p=0.832 |
| **Chest pain** | r=-0.156  p=0.342 | - | r=-0.141  p=0.475 |
| **Nausea** | r=-0.114  p=0.491 | r=-0.537  p=0.096 | r=0.061  p=0.758 |
| **Vomit** | r=-0.173  p=0.292 | - | r=-0.179  p=0.363 |
| **Abdominal pain** | r=-0.105  p=0.525 | r=-0.030  p=0.968 | r=-0.139  p=0.481 |
| **Flatulence** | r=0.127  p=0.441 | r=0.175  p=0.636 | r=0.130  p=0.510 |
| **Urge to defecate** | r=-0.009  p=0.959 | r=0.026  p=0.940 | r=-0.090  p=0.647 |
| **Diarrhoea** | r=-0.131  p=0.427 | - | r=-0.149  p=0.450 |
| **Stitches** | r=0.106  p=0.522 | r=-0.125  p=0.714 | r=0.194  p=0.323 |
| **Bloody diarrhoea** | **-** | **-** | **-** |
| **Total symptoms** | r=-0.166  p=0.307 | r=-0.169  p=0.617 | r=-0.114  p=0.557 |
| **Local exertion after 60min** | r=-0.123  p=0.443 | r=-0.459  p=0.156 | r=-0.013  p=0.944 |
| **Central exertion after 60 min** | r=0.129  p=0.422 | r=-0.217  p=0.519 | r=0.275  p=0.141 |
| **Local mean exertion** | r=-0.151  p=0.345 | r=-0.374  p=0.256 | r=-0.023  p=0.905 |
| **Central mean exertion** | r=0.169  p=0.292 | r=-0.032  p=0.929 | r=0.337  p=0.228 |

Correlations were performed by a two-tailed Spearman’s correlation analysis and corrected for multiplicity by the Benjamin-Hochberg procedure. p-values smaller than 0.05 and q-values smaller than 0.05 were considered statistically significant. L/R – urinary lactulose/rhamnose excretion ratio. Exertion assessed through the Borg RPE scale values. Mean exertion refers to the mean sum of all the values reported every 5 minutes through the exercise challenge.
